# PcsR2 Is a LuxR-Type Regulator That Is Upregulated on Wheat Roots and Is Unique to *Pseudomonas chlororaphis*

**DOI:** 10.3389/fmicb.2020.560124

**Published:** 2020-11-10

**Authors:** Huiqiao Pan, Leland S. Pierson, Elizabeth A. Pierson

**Affiliations:** ^1^Molecular and Environmental Plant Sciences Program, Texas A&M University, College Station, TX, United States; ^2^Department of Horticulture Sciences, Texas A&M University, College Station, TX, United States; ^3^Department of Plant Pathology and Microbiology, Texas A&M University, College Station, TX, United States

**Keywords:** plant-microbe interactions, interkingdom signaling, LuxR solos, *Pseudomonas*, phenazine

## Abstract

LuxR solos are common in plant-associated bacteria and increasingly recognized for playing important roles in plant-microbe interkingdom signaling. Unlike the LuxR-type transcriptional regulators of prototype LuxR/LuxI quorum sensing systems, *luxR* solos do not have a LuxI-type autoinducer synthase gene associated with them. LuxR solos in plant-pathogenic bacteria are important for virulence and in plant endosymbionts contribute to symbiosis. In the present study, we characterized an atypical LuxR solo, PcsR2, in the biological control species *Pseudomonas chlororaphis* 30–84 that is highly conserved among sequenced *P. chlororaphi*s strains. Unlike most LuxR solos in the plant-associated bacteria characterized to date, *pcsR2* is not associated with a proline iminopeptidase gene and the protein has an atypical N-terminal binding domain. We created a *pcsR2* deletion mutant and used quantitative RT-PCR to show that the expression of *pcsR2* and genes in the operon immediately downstream was upregulated ∼10-fold when the wild type strain was grown on wheat roots compared to planktonic culture. PcsR2 was involved in upregulation. Using a GFP transcriptional reporter, we found that expression of *pcsR2* responded specifically to root-derived substrates as compared to leaf-derived substrates but not to endogenous AHLs. Compared to the wild type, the mutant was impaired in the ability to utilize root carbon and nitrogen sources in wheat root macerate and to colonize wheat roots. Phenazine production and most biofilm traits previously shown to be correlated with phenazine production also were diminished in the mutant. Gene expression of several of the proteins in the phenazine regulatory network including PhzR, Pip (phenazine inducing protein) and RpeA/RpeB were reduced in the mutant, and overexpression of these genes *in trans* restored phenazine production in the mutant to wild-type levels, indicating PcsR2 affects the activity of the these regulatory genes upstream of RpeA/RpeB via an undetermined mechanism. Our results indicate PcsR2 upregulates the expression of the adjacent operon in response to unknown wheat root-derived signals and belongs to a novel subfamily of LuxR-type transcriptional regulators found in sequenced *P. chlororaphis* strains.

## Introduction

Albeit bacteria are unicellular organisms, they communicate with each other via small diffusible molecules to orchestrate their behaviors in a population density-dependent manner, which facilitates rapid adaptation to the environment (Fuqua et al., 1994; Mukherjee and Bassler, 2019). This cell-cell communication mechanism, known as quorum sensing (QS), enables populations to solve problems that single cells cannot, such as colonization, conjugation, secondary metabolites biosynthesis, biofilm formation, and effective invasion of host organisms (Piper et al., 1993; Fuqua and Winans, 1994; Pierson et al., 1994; Wood et al., 1997; Maddula et al., 2006; Schuster and Greenberg, 2006). In Gram- negative bacteria the diffusible signals are primarily fatty acid-derived whereas in Gram-positive bacteria they are most often derived from small peptides (Waters and Bassler, 2005; Pierson and Pierson, 2007). To date, QS systems utilizing *N*-acyl homoserine lactones (AHLs) are the most common in Gram-negative bacteria, although other types QS signals continue to be discovered (Schaefer et al., 2008; Ahlgren et al., 2011; Brachmann et al., 2013; Brameyer et al., 2015b; Corral-Lugo et al., 2016; Papenfort et al., 2017). The prototypical AHL-based QS system consists of a LuxI-type AHL synthase that produces an AHL signal and a cognate LuxR-type transcriptional regulator that senses the signal and regulates the expression of target genes, and autoinduction is a common feature of quorum sensing. Once the intracellular concentration of the signal exceeds a specific threshold, the signal binds to the LuxR, which in turn binds to a relatively conserved DNA sequence known as the *lux* box in the promoter region of regulated genes, activating or repressing their expression (Waters and Bassler, 2005). Quorum sensing was first identified in *Vibrio fischeri*, where LuxR binds the signal and directly activates transcription of the *luxICDABE* operon, resulting in an exponential increase in the production of both the signal and bioluminescence (Nealson and Hastings, 1979; Engebrecht et al., 1983; Fuqua and Greenberg, 2002).

Although LuxR transcriptional regulators were first characterized as components of QS systems, it is now recognized a majority of the annotated *luxR-*type genes do not have a *luxI* gene in proximity on the bacterial chromosome (Hudaiberdiev et al., 2015). This type of unpaired LuxR is referred to as a LuxR solo (or orphan LuxR) (Fuqua, 2006; Subramoni and Venturi, 2009a). Similar to the LuxRs of two-component QS systems, typical LuxR solo regulators consist of about 250 amino acids and have an autoinducer-binding domain at the N-terminus and a conserved helix-turn-helix (HTH) DNA-binding motif at the C-terminus. As reported previously, LuxR-type proteins exhibit low identities (18–25%), however, in most QS LuxR-type proteins nine amino acid residues involved in autoinducer-binding and DNA-binding are highly conserved (Gonzalez and Venturi, 2013). Although not universally accepted, the definition of LuxR solo has been expanded to include atypical LuxR solos, e.g., LuxR-type receptors that do not have a classic autoinducer domain (Brameyer et al., 2014; Hudaiberdiev et al., 2015). Atypical LuxRs solos characterized to date retain the HTH DNA-binding at the C-terminus, but have either Per-ARNT-Sim (PAS) signal-sensing domains, REC (receiver) signal receiver domains, or unidentified domains in place of the autoinducer-binding domain at the N-terminus (Wang et al., 2006; de Bruijn and Raaijmakers, 2009; Brameyer et al., 2014). In the present study, in keeping with the broad definition of LuxR solos, typical and atypical solo LuxR-family homologs will be included in our discussion of LuxR solos regardless of the domains at the N-terminus. LuxR solos are believed to expand the regulatory repertoire of the prototypical QS systems, wherein the LuxR solos or the expression of their encoding genes respond to exogenously produced AHLs, signal-mimics, or as yet unidentified interkingdom signals (Teplitski et al., 2000; Subramoni and Venturi, 2009b; Venturi and Fuqua, 2013; Martínez et al., 2015; Coutinho et al., 2018). It is believed that these widespread LuxR solos may mediate novel interkingdom communication, such as between symbiotic-living bacteria and their hosts (Subramoni and Venturi, 2009a; Gonzalez and Venturi, 2013; Brameyer et al., 2015a).

To date, several LuxR solos in plant-associated bacteria are known to play roles in plant pathogenesis or symbiosis, and some of these LuxR solos or the expression of their encoding genes respond to plant signals. The genes encoding these LuxR solos are usually located on the bacterial chromosome in close proximity to a proline iminopeptidase (*pip*) gene (Gonzalez and Venturi, 2013), which releases an N-terminal residue from a peptide, typically a proline. The promoter region of *pip* in all these strains contains a 20 bp inverted repeat sequence (*lux* box). XccR in the plant pathogen *Xanthomonas campestris* pv. *campestris* was the first LuxR solo reported to respond to unknown plant signals and both XccR and Pip are important for plant virulence (Zhang et al., 2007). Likewise, in other pathogenic bacteria, LuxR solos such as OryR in *Xanthomonas oryzae* pv. *oryzae* (Ferluga et al., 2007; Ferluga and Venturi, 2009; Gonzalez et al., 2013), XagR in *Xanthomonas axonopodis* pv. *glycines* (Chatnaparat et al., 2012), XocR in *Xanthomonas oryzae* pv. *oryzicola* (Xu et al., 2015), PsaR2 in *P. syringae* pv. *actinidiae* (Patel et al., 2014) responds to plant signals and are involved in bacterial pathogenicity, mobility, or host colonization. The genes encoding the LuxR solo NesR in the symbiotic plant-beneficial bacteria *Sinorhizobium meliloti* (Patankar and González, 2009) and PipR in the cottonwood endophyte *Pseudomomas* sp. GM79 (Schaefer et al., 2016) are also associated with *pip* genes and these LuxR solos regulate genes that contribute to symbiosis traits including plant nodulation and plant carbon source utilization, respectively.

In comparison to the aforementioned plant-pathogenic and plant-symbiotic bacteria, the genomes of many well-studied plant growth-promoting rhizobacteria (PGPR) contain numerous genes annotated as encoding solo LuxR-family homologs. For example the genomes of PGPR strains *P. chlororaphis* subsp. *aureofaciens* O6 and 30–84, *P. protegens* Pf-5, P. *brassicacearum* Q8r1-96, *P. fluorescens* Pf0-1, Q2-87, SBW25, A506, SS101 and *P. synxantha* BG33R (Loper et al., 2012) contain 12–28 solo *luxR-*type genes based on gene annotations in the National Center for Biotechnology Information (NCBI) database^1^. Also, in comparison to the aforementioned examples, the context of the genes encoding the LuxR solos studied in PGPR thus far present distinctive features. For example, the promoter of the *pip* gene adjacent to *psoR* in *P. protegens* CHA0 and *P. protegens* Pf-5 lacks a *lux*-box palindrome (Subramoni et al., 2011) and there is no *pip* adjacent to *lesR* in *L. enzymogenes* (Qian et al., 2014) or *viscAR* and *viscBCR* in *P. fluorescens* SBW25 de Bruijn and Raaijmakers, 2009). LuxR-type regulators ViscAR and ViscBCR have a HTH DNA-binding domain but lack a classic autoinducer domain. All these LuxR solos activate genes important for biological control activity. For example, PsoR activates anti-microbial-related genes in response to plant signals from wheat and rice but not cucumber. LesR activates the production of heat-stable antifungal factor (HSAF) and ViscAR and ViscBCR regulate viscosin production important for antagonism of oomycete plant pathogens.

Based on these few examples, we hypothesize LuxR solos may enable PGPR to sense and respond to interkingdom signals, facilitating bacterial adaptation to a symbiotic lifestyle and the expression of traits important for plant growth-promoting activities. In the present study, we examined the LuxR-type regulators in the well characterized PGPR strain *Pseudomonas chlororaphis* subsp. *aureofaciens* 30–84 (hereafter *P. chlororaphis* 30–84). This strain was selected as a biological control strain for take-all disease of wheat and was isolated from the roots of wheat grown in a take-all disease suppressive soil (Pierson and Thomashow, 1992; Pierson et al., 2013). Production of phenazines, heterocyclic nitrogen-containing secondary metabolites produced by *P. chlororaphis* 30–84 were shown to be the primary mechanism of disease suppression (Pierson et al., 1994; Pierson and Pierson, 1996). Phenazine production by *P. chlororaphis* 30–84 is necessary for effective inhibition of the take-all pathogen *Gaeumannomyces graminis var. tritici* (Pierson and Thomashow, 1992), persistence of *P. chlororaphis* 30–84 in the wheat rhizosphere (Mazzola et al., 1992), and biofilm formation (Maddula et al., 2006), and wheat seedling drought tolerance (Mahmoudi et al., 2019). Phenazines are produced by a diversity of prokaryotes and have been shown to play roles in Reactive Oxygen Species (ROS) generation, electron shuttling, and iron chelation in other *Pseudomonas* species (Pierson and Pierson, 2010; Wang et al., 2010, 2011; Recinos et al., 2012; Das et al., 2015). Phenazine biosynthesis is controlled by a complex network of regulatory genes organized in a hierarchical manner (Pierson et al., 1998; Whistler and Pierson, 2003; Pierson and Pierson, 2010; Wang et al., 2012, 2013; Yu et al., 2018). The heart of this network is control by the LuxR-type transcriptional regulator (PhzR), part of the PhzR/PhzI QS system that directly regulates phenazine biosynthesis (Pierson et al., 1994; Wood and Pierson, 1996). A second set of QS genes CsaR/CsaI, indirectly influences phenazine production (Zhang and Pierson, 2001).

In our survey for potentially plant-responsive LuxR solo candidates in the genome of *P. chlororaphis* 30–84, we identified one atypical LuxR solo (PcsR2) based on the observation that *pcsR2* expression was highly upregulated when bacteria were grown on wheat roots compared to planktonic culture. We provide evidence that the expression of *pcsR2* and the adjacent operon respond to wheat root-derived signals rather than endogenous AHLs, and that PcsR2 is required for this response. We describe bacterial phenotypes modulated by PcsR2. PcsR2 is involved in the expression of bacterial traits that contribute to the utilization of carbon and nitrogen from wheat roots, phenazine biosynthesis, and biofilm production, traits that promote the plant-associated lifestyle and plant growth promoting activity of the bacteria. We discuss the novelty of PcsR2 in relation to other known plant responsive LuxR solos.

## Materials and Methods

### Bacterial Strains, Plasmids, and Growth Conditions

Bacterial strains and plasmids used in this study are described in Table 1, and primers are listed in Supplementary Table 1. A spontaneous rifampicin-resistant derivative of *P. chlororaphis* 30–84 was used in all studies and is hereafter referred to as 30–84WT. *P. chlororaphis* was grown at 28°C in Luria-Bertani (LB) medium (Fisher BioReagents^TM^, Hampton, NH), pigment production medium D (PPMD), AB minimal media (AB), AB amended with 0.4% glucose (AB + G), or AB + G amended 2% casamino acids (AB + CAA) media (CAA is from BD Bacto, San Jose, CA), as described previously (Yu et al., 2018). *Escherichia coli* (*E. coli*) was grown at 37°C in LB medium. *E. coli* and *P. chlororaphis* were grown in liquid culture with agitation (200 rotations per minute) or on solid medium (amended with agar at 15 g/l). Antibiotics were used in the following concentrations for *E. coli:* kanamycin (Km), gentamicin (Gm), ampicillin (Ap), 5-bromo-4-chloro-3-indolyl-β-D-galactopyranoside (X-gal) at 50, 15, 100, 40 μg/ml, respectively; and for *P. chlororaphis:* Km, Gm, Ap, rifampicin (Rif), Cycloheximide (Cyclohex) at 50, 50, 100, 100, 100 μg/ml, respectively.

**TABLE 1:** Strains and plasmids used in this study.

Strains and plasmids	Descriptions	References
***P. chlororaphis***		
30–84WT	Phz^+^, Rif^R^, wild-type (WT)	Whistler and Pierson, 2003
30–84ΔpcsR2	Km^R^, *pcsR2* replaced with Km^R^ cassette	This study
30–84ΔpcsR2(pGT2PcsR2)	Complemented mutant containing plasmid pGT2PcsR2	This study
30–84WT(pGT2PcsR2)	WT with plasmid pGT2PcsR2	This study
30–84ZN	Phz^–^, Rif^R^, *phzB*:*lacZ* genomic fusion	Wood et al., 1997
30–84R	Phz^+^ Rif^R^ *phzR:Tn5* genomic fusion, Km^R^	Pierson et al., 1994
***E. coli***		
DH5α	F*^–^rec*A1 *end*A1 *hsdR*17 *sup*E44 *thi*-1 *gyr*A96 *rel*A1 Δ*(arg*F*-lac*ZYA*)* I169 Φ80lacZΔM15λ^–^	GIBCO-BRL
***Plasmid***		
pGT2PcsR2	Promoter *tac:pcsR2* fusion in pGT2P created via replacement of *lacZ* with *pcsR2* in pGT2Ptac:lacZ	This study
pGT2PpcsR2:gfp	pGT2Ptac:lacZ with promoter of the *pcsR2* operon replacing Ptac promoter	This study
pGT2P4806:gfp	pGT2Ptac:lacZ with operon 2 promoter replacing Ptac	
pGT2Ptac:lacZ	pGT2 containing a constitutive promoter p*tac:lacZ* fusion (Ptac promoter drives *lacZ* and *gfp* expression)	Yu et al., 2017
pGT2	pProbe-GT’: pVS1 replicon, p15a origin of replication, *gfp* transcriptional fusion; Gm^R^	Miller et al., 2000
pEX18Ap	Ap^R^	Hoang et al., 1998
pUC4K	Km^R^, Ap^R^	Grindley and Joyce, 1981
pGT2Ptac:phzR	*lacZ* in pGT2Ptac:lacZ is replaced with 0.9 kb DNA fragment containing *P. chlororaphis* 30–84 *phzR*	Wang et al., 2013
pUCRpeB	1.2 kb DNA fragment containing *rpeB* in pUCP20G	Yu et al., 2017
pUCPip	784 bp DNA fragment containing *pip* in pUCP20G	Yu et al., 2017
pUCRpoS	1.4 kb DNA fragment containing *rpoS* in pUCP20G	Yu et al., 2017
pUCP20G	Gm^R^, pUCP20 derivative containing constitutive promoter pLac with *Sma*I-flanked Gm^R^ cassette inserted into the unique *Sca*I site within *bla*	Chiang and Burrows, 2003

*Ap^R^, Km^R^, Gm^R^, Rif^R^, indicate ampicillin, kanamycin, gentamicin, rifampin, respectively.*

### Phylogenetic Analysis of PcsR2

LuxR-family homologs within the *P. chlororaphis* 30–84 genome were identified based on annotation (GenBank: CM001559.1) in the National Center for Biotechnology Information (NCBI) database^2^. The amino acid sequences *P. chlororaphis* 30–84 LuxR homologs and other characterized LuxR sequences were retrieved from the *Pseudomonas* database^3^ or NCBI. Protein domain analysis was performed using the NCBI conserved domain search^4^ and Pfam^5^. Sequence alignments and amino acid identity comparisons were performed using Clustal-omega^6^. Representative LuxRs were displayed graphically using Boxshade^7^. A maximum likelihood (ML) phylogenetic tree was constructed from multiple-sequence alignments of PcsR2 homologs in 48 fully sequenced strains of *P. chlororaphis* with MEGA7 (Kumar et al., 2016) using MUSCLE (MUltiple Sequence Comparison by Log- Expectation). The Jones, Taylor, and Thorton (JTT) model in MEGA 7 and bootstrap analysis with 1,000 replicates were used. Values greater than 50 are indicated at the nodes.

### Measuring Gene Expression Levels on Wheat Roots

The four solo LuxR homologs with the fewest number of substitutions among the nine highly conserved amino acid residues involved in signal-binding and DNA-binding (Gonzalez and Venturi, 2013) were selected for testing (Table 2). Quantitative reverse transcription polymerase chain reaction (qRT-PCR) was used to compare the expression levels of the encoding genes when bacteria were grown in planktonic culture or on wheat roots. For the planktonic culture treatment, bacteria were grown in AB + CAA to a standard optical density (OD_620_ = 1.0, 28°C, with agitation). A 1 ml volume of the culture was mixed with 2 ml of Qiagen RNA Protect reagent (Qiagen, Hilden, Germany) to stabilize bacterial RNA. Bacterial cells were collected by centrifugation (16,000 × g). Whole RNA was extracted, and cDNA was obtained as described below. For the wheat root treatment, the hard red winter wheat cultivar TAM 112 (Reddy et al., 2014; Rudd et al., 2014) was used for all studies. Wheat seeds were surface-disinfested and grown in CYG^TM^ (Mega International, Newport, MN) germination pouches (in the dark, 28°C, receiving 10 ml distilled water every 2 days) until the roots were 10–15 cm in length (6–10 days). Bacterial inoculum was prepared by growing bacteria in AB + CAA to a standard optical density (OD_620_ = 1.0, at 28°C, with agitation). Plant roots were immersed in bacterial inoculum for 10 min, and then inoculated plants were transferred to modified germination pouches (roots wrapped in a single moistened sheet of germination paper) and grown for 16 h (8 h light/8 h dark, 28°C). The entire root system (6 plants/rep) was rinsed with 5 ml of Qiagen RNA Protect reagent (Qiagen, Hilden, Germany) to stabilize bacterial RNA and collect the bacteria. The cells were collected by centrifugation (1,200 × g). Total RNA was extracted using a Qiagen RNeasy Mini Kit (Qiagen) according to the manufacturer’s recommended protocol. The genomic DNA was removed using on-column DNase-I digestion (Qiagen) for 15 min. RNA concentration and purity were determined using a GE NanoVue spectrophotometer (GE Healthcare, Pittsburgh, PA). Total RNA (1 μg) was reverse transcribed using random primers (Promega, Madison, WI) and AMV Reverse Transcriptase (Promega, Madison, WI) according to the manufacturer’s protocol.

**TABLE 2:**
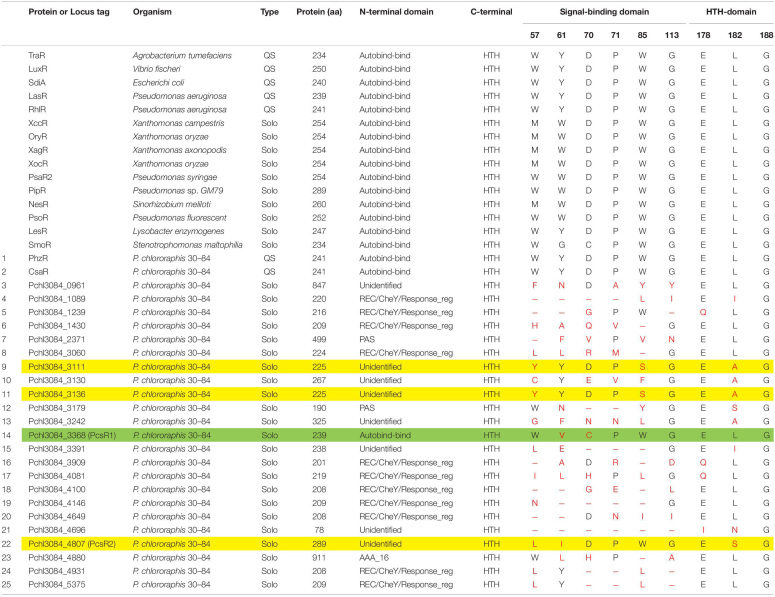
Representative LuxR regulators and LuxR-family regulators annotated by NCBI in the genome of *Pseudomonas chlororaphis* 30–84 and their protein characteristics.

*Representative QS LuxR and LuxR solos appear at the top of the table and the 25 solo LuxR-family homologs identified by annotation from P. chlororaphis 30–84 are numbered at the bottom of the table and referred to by their locus tag. N-terminal domains are identified using Pfam database nomenclature. The nine conserved amino acids in the signal binding domain and the helix-turn-helix (HTH) domains are indicated (numbering relative to TraR). Amino acid substitutions are indicated in red and red dashes indicate no amino acids aligned at that position. One P. chlororaphis 30–84 solo LuxR homolog has a typical autoinducer binding domain (PcsR1, highlighted in green). Highlighted in yellow are atypical candidate LuxR solos in P. chlororaphis 30–84 with only three amino acids substitutions among the nine conserved amino acids.*

A second experiment compared the expression levels of the *pcsR2* and two genes in the operon immediately downstream of *pcsR2* (Pchl3084_4801, Pchl3084_4803) in 30–84WT, 30–84ΔpcsR2, and 30–84ΔpcsR2 (pGT2PcsR2) when bacteria were grown in planktonic culture or on wheat roots using qRT-PCR as described above.

SYBR Green reactions were performed using the CFX384^TM^ Real-Time System (Bio-Rad, Hercules, CA) in Optical 384-Well Reaction 1Plates (Applied Biosystems, Foster City, CA). Quantitative PCR (qPCR) assays were performed to measure the expression levels of the target genes as previously described with minor modifications (Yu et al., 2018). Briefly, synthesized cDNA (5 ng/reaction) or a negative control was used for qPCRs with Fast SYBR Green^®^ PCR Master Mix (Thermo Fisher Scientific, Waltham, MA) and gene-specific primers (250 nM final concentration). qPCR amplification was used to detect the expression of the four *luxR* solo genes in the planktonic and wheat root growth treatments. Expression of *rpoD* was used as the housekeeping control. qPCR amplifications were carried out at 50°C for 2 min, 95°C for 10 min, followed by 40 cycles of 95°C for 15 s and 60°C for 1 min, and a final dissociation curve analysis step from 65 to 95°C. Amplification specificity for each reaction was confirmed by dissociation curve analysis. The relative expression of the target gene was determined based on the mean of cycle threshold (Ct) values and ΔΔCt was calculated by normalizing target gene expression to the expression of *rpoD* as the reference gene. All values are the means of three replicates. The primers for qRT-PCR are listed in Supplementary Table 1.

### Generation of *pcsR2* Mutant, Complementation, and Overexpression

#### Mutant Cloning Strategy

A derivative of 30–84WT containing a *pcsR2-*deletion mutation was generated using strategies and methods described previously (Hmelo et al., 2015) with minor modifications. Briefly, fragments containing the upstream and downstream coding sequences (459 base pair each) flanking *pcsR2* were amplified by PCR using the primer pairs 4807KO-UP-F-*Eco*RI (4807KO-1) and 4807K0-UP-R-*Pst*I (4807KO-2), and 4807KO-DN-F-*Pst*I (4807KO-3) and 4807KO-DN-R-*Hin*dIII (4807KO-4), respectively. The two amplicons each have 24 nt overlap at 3′ and 5′ end, respectively, due to the overlap of 4807KO-2 and 4807KO-3. Using the primers 4807KO-1 and 4807KO-4 and the products of the previous PCR as a template, overlap PCR was performed. The new product was a DNA fragment that fused the upstream and downstream coding sequence of *pcsR2* with a *Pst*I site at their junction. This fragment was ligated into the *Eco*RI and *Hin*dIII restriction enzyme sites in the multiple-cloning region of the suicide vector pEX18Ap. The modified plasmid was then transformed into *E. coli* and screened via blue-white color on LB with ampicillin and Xgal. A Km resistance cassette with its promoter (916 bp) was amplified from pUC4K using primer pairs KmPstI-F and KmPstI-R. Following *Pst*I digestion, the resistance cartridge was inserted into the *Pst*I site of the modified pEX18Ap construct via T4 ligation. The final construct was conjugated into 30–84WT, and positive transformants were selected on LB (amended with Km and Rif). A double-crossover mutant (30–84ΔpcsR2) was obtained by counter-selection with LB amended with Km and 6% sucrose and checked by PCR primers 4807KO-UP-UP-F(4807Check1), 4807-DN-DN-R(4807Check2), KmPstI-F, 4807qPCR-R PCR was performed using FideliTaq^TM^ DNA Polymerase (Affymetrix, Santa Clara, CA) or GoTaq^®^ Green Master Mix (Promega, Madison, WI) according to manufacturer instructions. Vector constructions were verified via Sanger sequencing using an ABI 3130xl Genetic Analyzer (Laboratory for Genome Technology, Texas A&M University). *E. coli* transformation and *P. chlororaphis* conjugation were performed as described previously (Pierson and Thomashow, 1992; Wang et al., 2012).

#### Strategy for Complementation and Overexpression

For complementation of 30–84ΔpcsR2, a plasmid constitutively expressing *pcsR2* via the P*tac* promoter was created. This was done by replacing the coding sequence of *lacZ* with *pcsR2* in the expression vector pGT2Ptac:lacZ. The coding sequence of *pcsR2* was PCR amplified with the primers 4807-F-BamH1 and 4807-R-*Hin*dIII. The resulting fragment was digested with *Bam*HI and *Hin*dIII and cloned into the GT2Ptac:lacZ expression vector creating a *Ptac:pcsR2* fusion, and the plasmid (pGT2PcsR2) was introduced into *E. coli*. The sequence was confirmed by Sanger sequencing. The expression vector was transformed to 30–84ΔpcsR2 by conjugation to generate 30–84ΔpcsR2(pGT2PcsR2), the complemented version of the mutant. A *pcsR2* overexpression derivative also was obtained by conjugating the plasmid into 30–84WT to generate 30–84WT(pGT2PcsR2). For all comparisons utilizing the complement strain, 30–84WT and 30–84ΔpcsR2 containing the plasmid with no insert (NI), e.g., 30–84WT(NI) and 30–84ΔpcsR2(NI) were used. Standard growth curves were obtained for strains grown in AB + CAA at 28°C, with agitation.

### Effect of AHLs and Plant Macerate on Gene Expression and Bacterial Growth

AHLs were extracted from 30 to 84ZN (lacks phenazine production) in ethyl acetate and quantified as described previously (Whistler and Pierson, 2003). Wheat macerate was extracted as described previously, with minor modification (Schaefer et al., 2016). Briefly, wheat seeds (variety TAM 112) were surface disinfected and grown in growth pouches as described above. After 6–10 days, 2.5 g of roots or leaves were macerated in the presence of liquid nitrogen and resuspended in 100 ml distilled water, which then was filtered to remove plant tissue.

A reporter of *pcsR2* transcription (pGT2PpcsR2:gfp) was constructed by PCR amplification of the promoter sequence of the *pcsR2* operon (Pchl3084_4808-4807) using primers 4808pr-*Eco*RI-F and 4808pr-*Bam*HI-R. The PCR product was then ligated into the *Eco*RI and *Bam*HI sites replacing the Ptac promoter within the reporter vector pGT2Ptac:lacZ (thus the promoter drives both *lacZ* and *gfp* expression, Table 1). Similarly, a reporter of operon 2 transcriptional activity (pGT2P4806:gfp) was constructed by PCR amplification of the promoter sequence of operon 2 (Pchl3084_4806-4800) using primers 4806pr-*Eco*RI-F and 4806pr-*Bam*HI-R. The reporters then were introduced into 30–84WT and 30–84ΔpcsR2 by conjugation.

The GFP reporter was used to determine whether certain plant extracts activated gene expression as described previously (Schaefer et al., 2016) with minor modifications. Strains containing reporter plasmid pGT2PpcsR2:gfp were grown overnight in AB + G (AB minimal media supplemented 0.4% glucose) at 28°C with agitation and cells were sub-cultured 1:10 into fresh AB + G supplemented with and without AHLs or different concentrations (1, 10, 20% v/v) of root macerate (RM) or leaf macerate (LM) in individual wells of a 96-well microtiter. The plates were incubated at 28°C with minimal agitation (10 s mixing, 3 times every 3 h). GFP intensity (excitation at 485 nm and emission at 535 nm) and cell density (OD_620_) were measured using Tecan Infinite M200 Pro with I-control software (Tecan, Mannedorf, Switzerland) at 12 h. Strains carrying the no-insert plasmid were used as the blank. Data were plotted as relative fluorescence units (RFU) per OD unit. The β-galactosidase activity of the reporter was also used to verify gene expression levels as described previously (Yu et al., 2018).

To determine whether wheat root macerate could be used as the primary carbon or nitrogen source, four types of modified bacterial media were prepared: AB without glucose as the carbon source (AB-C), AB without NH_4_Cl and as the nitrogen source of (AB-N), AB-C media supplemented with 80% root macerate (v/v) as the carbon source (AB-C + M), and AB-N media supplemented with 80% root macerate (v/v) as the nitrogen source (AB-N + M). The three strains 30–84WT, 30–84ΔpcsR2, and 30–84ΔpcsR2 (pGT2PcsR2) were grown separately overnight in AB + G and the cells were collected via centrifugation (16,000 × g), washed three times, and resuspended in sterile distilled water. The final cell density was standardized to OD_620_ = 0.8. The bacterial cultures were inoculated separately at a 1:100 dilution into a 96 well plate. After 24 h (28°C, with agitation), cell density (OD_620_) was measured. A minimum of three replicates per experiment were used and experiments were repeated at least twice.

### Root Persistence Assay

Root persistence assays measuring the populations of 30–84WT or 30–84ΔpcsR2 on roots were conducted as described previously with minor modification (Mazzola et al., 1992; Dorosky et al., 2018). Soil used for these experiments was a Pullman clay loam collected from the USDA-ARS, Bushland, TX dryland wheat plots at a depth of 1–15 cm. Prior to use, it was necessary to sieve (2 mm mesh) and mix the soil with sand (soil: sand, 2:1, v:v) to facilitate drainage. The soil-sand mix, hereafter referred to as soil, was autoclaved twice (121°C, 15 PSI, 1, 24 h pause between cycles). For bacterial inoculum, 30–84WT and 30–84ΔpcsR2 were grown AB + CAA, collected via centrifugation (16,000 × g), washed, and resuspended in distilled water to a final OD_620_ = 1.0. Aliquots of the cell suspension (2 ml) were diluted with distilled water (18 ml) and added to 500 g of soil and mixed thoroughly. Soil was dispensed into plastic tubes (Ray Leach Cone-tainers, 2.5 cm diameter ×16.5 cm, 10 g soil/tube). Prior to planting, wheat seeds (TAM112) were surface disinfested and germinated on sterilized germination paper. Two-day-old seedlings were planted into each container and covered with autoclaved vermiculite 4 days after the bacteria were inoculated into the soil. Each treatment had 10 replicate plants and soil without bacterial inoculation was used as the control. After 6 weeks of growth (8 h dark/16 h light cycle at 28°C), the entire root system (and loosely adhering soil) was collected and immersed in 10 ml of phosphate-buffered saline (PBS) solution, and bacteria were collected by sonicating and vortexing. Bacterial populations were enumerated via serial dilution plating onto media (amended with Rif and Cyclohex). CFU was calculated after 48 h and normalized to root dry weight (48 h, 65°C).

### Growth, Phenazine, and Biofilm Quantification

For growth curves in AB + CAA, LB, PPMD, populations were quantified spectrophotometrically (at OD_620_) using an Opsys MR Microplate Reader (Thermo Fisher Scientific, Waltham, MA) or via serial dilution plating and colony counting (CFU/ml). Phenazines were extracted as described previously (Wang et al., 2016). Phenazines were quantified by absorbance at OD_367_ using a Nanovue plus (GE Healthcare Life Sciences, Pittsburgh, PA). The absorbance for each sample was normalized to the total absorbance of 10 ml cultures.

Surface attached biofilms of cultures grown in 96 well plates were quantified using the crystal violet method as described previously (Maddula et al., 2006) and biofilm populations were quantified spectrophotometrically (OD_540_). The concentration of extracellular (eDNA) present in floating biofilms was quantified (μg/ml) using a Qubit dsDNA Assay Kit and Qubit 2.0 Fluorometer (Invitrogen, Carlsbad, CA) according to the manufacturer’s instructions, as described previously (Wang et al., 2012). Matrix from floating biofilms of cultures grown in 24-well plates was collected and quantified as previously described with minor modification (Wang et al., 2016). At the time of sampling, the entire culture (including floating biofilm) was transferred to 1.5 ml Eppendorf tubes and biofilm matrix was collected by centrifugation (16,000 × g for 5 min). The supernatants were removed, and the mass of cells and hydrated matrix weighed. For DNase treatment, 30 units of water-dissolved DNase I (Qiagen) were added to 24 h bacterial cultures and then incubated an additional 24 h. The biofilm matrix was quantified as above.

### qRT-PCR of Regulators of Phenazine Production

Expression levels of *phzI*, *phzR*, *rpeB*, *pip* (phenazine inducing protein, not proline iminopeptidase), and *rpoS* were measured via qRT-PCR in 30–84WT(NI), 30–84ΔpcsR2(NI) and 30–84ΔpcsR2(pGT2PcsR2). The protocol for extraction of whole RNA and qRT-PCR was as described above. The primers for qRT-PCR are listed in Supplementary Table 1. Gene expression levels were expressed as ΔΔCt, relative to *rpoD*.

### Constitutive Expression of *phzR*, *rpeB*, *pip*, and *rpoS* in *pcsR2* Mutant

The previously constructed plasmids pGT2PhzR, pUCPip, pUCRpeB, and pUCRpoS with constitutive promoters were introduced into 30–84WT and 30–84ΔpcsR2 by either electroporation or triparental conjugation. After 48 h incubation, phenazine production was quantified as described above. 30–84WT(NI) and 30–84ΔpcsR2(NI), with the empty vector (pGT2 or pUCP20) were used as controls. A minimum of three replicates per experiment were used and experiments were repeated at least twice.

### Statistical Analysis

All data presented are mean ± the standard error from at least two experiments. Multiple comparisons were analyzed using ANOVA and Turkey HSD and significant differences (*P* < 0.05) are indicated by lowercase letters. Two-group comparisons (WT versus mutant) were performed using Student’s *t*-test and asterisks indicate significant differences (*P* < 0.05). All data were analyzed using JMP Version 14 Software (SAS Institute In., Cary, NC).

## Results

### Identification of a Novel LuxR Solo With Elevated Gene Expression on Roots

Twenty-five genes are annotated by NCBI as encoding LuxR-family transcriptional regulators in the *P. chlororaphis* 30–84 genome, including those encoding the two previously described QS LuxRs (PhzR and CsaR). The other twenty-three solo LuxR-type homologs we refer to by their locus tag and are listed in Table 2. Protein domain analysis indicated that one of the solo LuxR homologs (Pchl3084_3368) is a typical LuxR solo, i.e., having an autoinducer-binding domain (PFAM03472) at the N-terminus (hereafter PcsR1, Table 2). The other twenty-two LuxR-type homologs possess a helix-turn-helix (HTH) DNA-binding domain (PF00196) at the C-terminus, typical of LuxR-family regulators. However, they are atypical in that the N-terminal domains lack autoinducer-binding domains and instead have either PAS signal-sensing, REC/CheY signal receiver, AAA ATPase domains or unidentified domains in place of the autoinducer-binding domains (Table 2). Several of the solo LuxR-family homologs have predicted protein lengths that are considerably smaller than or greater than known LuxR solos (Pchl3084_4696, Pchl3084_0961, and Pchl3084_4880). The typical LuxR solo (Pchl3084_3368) has two substitutions among nine highly conserved amino acid residues. Three of the atypical LuxR solos (Pchl3084_4807, Pchl3084_3111, Pchl3084_3136) have three substitutions among the nine and the others have as many as eight. In our survey for potentially plant-responsive LuxR solo candidates, we focused first on the four homologs with the fewest amino acid substitutions among the conserved amino acids involved in the signal-binding and DNA-binding domains (Table 2).

In a comparative analysis, relative gene expression (ΔΔCt, measured using qRT-PCR standardized to *rpoD*) of one of the four solo LuxR homologs (Pchl3084_4807) was highly upregulated when 30–84WT was grown on wheat roots compared to growth in planktonic culture (9.5 ± 1.4 vs. 1.0 ± 0.1, respectively). In contrast, differences in the gene expression of the other three LuxR homologs were not as pronounced (data not shown). Based on gene expression patterns, Pchl3084_4807, hereafter referred to as PcsR2 became the focus of subsequent work. The *pcsR2* coding sequence is 870 nucleotides in length and thus predicted to encode a LuxR-type regulator somewhat larger than a typical LuxR (e.g., 289 amino acids vs. 250, respectively), but the same size as the LuxR solo PipR in the cottonwood endophyte *Pseudomonas* sp. GM79 (Schaefer et al., 2016).

### PcsR2 Is a Member of a Novel Subfamily of LuxR-Type Transcriptional Regulators Conserved in *P. chlororaphis*

**FIGURE 1:**
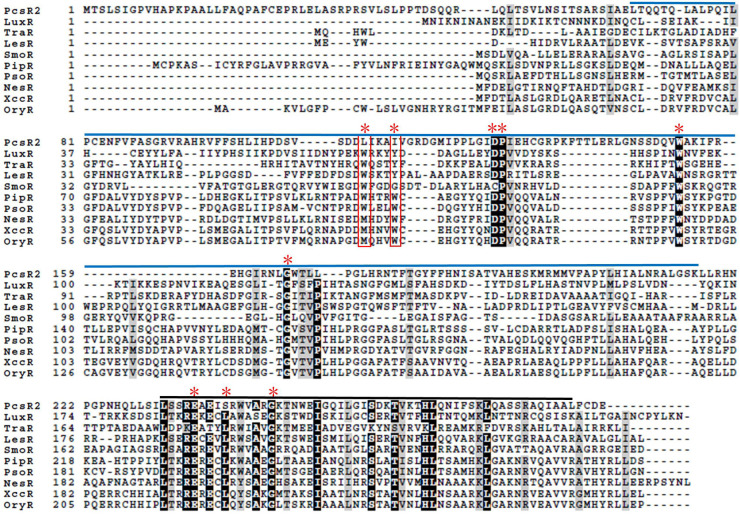
Amino acid sequence alignments of PcsR2 and other representative LuxR-type transcriptional regulators. Sequence alignments and amino acid identity comparisons were performed using Clustal-omega, and Boxshade was used to highlight the degree of amino acid identity (90–100%). The blue and black lines above the sequences indicate the signal-binding and helix-turn-helix domains of QS LuxRs, respectively. Red asterisks indicate the positions of nine conserved amino acids in QS LuxRs. The two residue positions boxed in red are the most common residues that differ between QS LuxRs (such as LuxR and TraR) and the LuxR solo homologs from plant-associated species (such as PipR, PsoR, NesR, XccR, and OxyR). The PcsR2 sequence also varies in these two positions (L_57_, I_61_ instead of W_57_, Y_61_ in canonical LuxRs; positions numbered as in TraR). Included in this table with PcsR2 are QS LuxR homologs: LuxR in *Vibrio fischeri* and TraR in *Agrobacterium tumefaciens*, and LuxR solos: LesR in *Lysobacter enzymogenes;* SmoR in *Stenotrophomonas maltophilia*; PipR in *Pseudomonas* sp. strain GM79; PsoR in *P. fluorescens and P. protegens*; NesR in *Sinorhizobium meliloti*; XccR in *Xanthomonas campestris* pv. *campestris;* OryR in *X. oryzae* pv. *oryzea.*

Analysis of conserved LuxR protein domains of PcsR2 indicated that it possesses a typical HTH DNA-binding domain (PF00196) at the C-terminus, but no AHL-binding domain (PFAM03472) or any other conserved binding domain at the N-terminus. Amino acid sequence alignment of PcsR2 with other representative LuxR transcriptional regulators was used to compare the nine highly conserved amino acids (red asterisk) within the N-terminal signal-binding and C-terminal HTH domains (Figure 1, indicated by blue vs. black lines, respectively and Table 2). PcsR2 has one substitution out of three highly conserved residues in the HTH domain. Despite the lack of an AHL-binding domain, PcsR2 retains four of six highly conserved amino acid residues within the signal binding domain, which are also conserved in known QS LuxRs (such as LuxR and TraR) as well as previously published plant-responsive LuxR solos such as LesR, PipR, PsoR, NesR, XccR, and OryR. The differences between PcsR2 and the other LuxR solos in the substitutions in the conserved amino acid residues within the signal-binding domain suggest that PcsR2 may respond to different signals than those reported previously for plant-responsive LuxR solos.

Analysis of the *pcsR2* gene context indicated that different from previously characterized *luxR* solo genes such as *oryR, xccR, xocR*, or *xagR* in plant-pathogenic strains and *pipR*, or *psoR* in plant-beneficial strains (Gonzalez and Venturi, 2013), the *pcsR2* locus is not associated with a proline iminopeptidase (*pip*) gene (although there is a gene locus annotated as encoding this protein elsewhere in the genome (Pchl3084_0411), it is not associated with a transcriptional regulator). Instead, *pcsR2* is part of a two gene operon with an AMP-binding protein belonging to a family of adenylate-forming enzymes that generate an acyl-AMP intermediate (Supplementary Figure 1). Members of this family include long chain fatty acid-CoA ligase, acetyl-CoA synthetase, and various other closely related synthetases. Located downstream is an operon containing two genes annotated as being involved in fatty acid desaturation. PcsR2 bears low amino acid identity with other well-characterized QS LuxR proteins such as LuxR (22%), TraR (15%) or even PhzR in *P. chlororaphis* 30–84 (18%) or LuxR solos such as LesR (20%), PipR (20%), and PsoR (20%) in other plant-associated bacteria. By contrast, it bears extremely high amino acid sequence identity (96–100%, over the entire amino acid sequence) with PcsR2 homologs found in other sequenced strains of *P. chlororaphis* (Supplementary Table 2) and the organization of these LuxRs in operons with an AMP-binding protein is conserved. Interesting based on amino acid sequence similarity, the PcsR2 homologs in the sequenced *P. chlororaphis* strains generally cluster by subspecies (Supplementary Figure 2).

In support of the hypothesis PcsR2 is a LuxR-type transcriptional regulator of adjacent operon 2 and its own operon, we identified putative *lux* box homologs in the promoter regions of both operons. In operon 2, the putative *lux* box sequence is centered 73 bp upstream of translation start codon and in operon 1 it is centered 80 bp upstream of the translational start codon (Supplementary Figure 1). Together, these results are consistent with the hypothesis that PcsR2 is a member of a novel subfamily of LuxR-type transcriptional regulators that is found thus far only in *P. chlororaphis.*

### Expression of *pcsR2* and the Downstream Operon Are Upregulated on Wheat Roots

**FIGURE 2:**
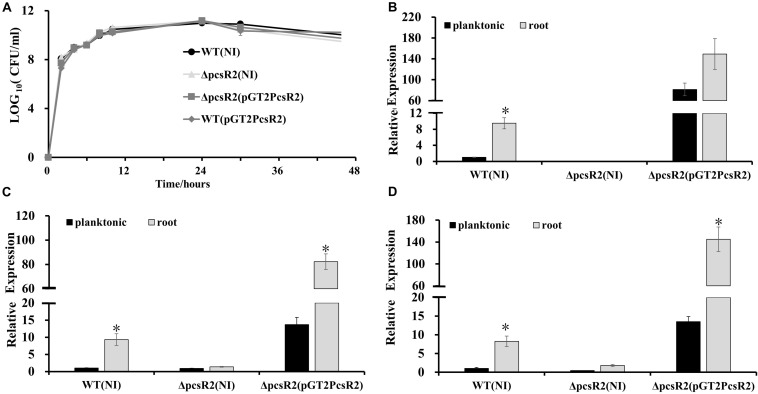
Expression of *pcsR2* and the downstream operon were upregulated on wheat roots. **(A)** Growth curves of strains grown in AB + CAA at 28°C, for 48 h, with agitation. Populations were enumerated via serial dilution and colony counts (CFU). **(B–D)** Expression of *pcsR2* and two genes in operon 2 (Pchl3084_4801, Pchl3084_4803) in 30–84WT, 30–84ΔpcsR2, or 30–84ΔpcsR2(pGT2PcsR2) when strains were grown on wheat roots as compared to within planktonic culture (AB + CAA) for 16 h. Expression levels were measured via qRT-PCR (where relative gene expression is expressed as ΔΔCt, with *rpoD* as internal control) and data are the mean and standard error of nine replicates. Gene expression levels were compared by strain using a Student’s *t*-test. Asterisks indicate whether treatments are significantly different (*P* < 0.05).

A derivative of 30–84WT containing a *pcsR2-*deletion mutation (30–84ΔpcsR2) and the mutant complemented via constitutive expression (30–84ΔpcsR2(pGT2PcsR2)) were used to verify *pcsR2* response to plant signals from roots (Table 1). For all comparisons utilizing the complement, 30–84WT and 30–84ΔpcsR2 containing the plasmid with no insert (NI), e.g., 30–84WT(NI) and 30–84ΔpcsR2(NI) were used. All strains grew similarly to each other in planktonic culture in AB + CAA and reached stationary phase by 24 h (Figure 2A). The relative gene expression of *pcsR2* (ΔΔCt, measured using qRT-PCR standardized to *rpoD*) was highly upregulated when 30–84WT was grown on wheat roots as compared to within planktonic culture in AB + CAA. In contrast, there was almost no expression of *pcsR2* in the mutant in either condition, indicating the specificity of the primers. The relative gene expression of *pcsR2* in 30–84ΔpcsR2(pGT2PcsR2) was greater than 30–84WT in both conditions due to the higher copy number and constitutive expression of *pcsR2*, but the fold difference in gene expression in the two conditions was less (Figure 2B). These results verified that *pcsR2* is upregulated when 30–84WT is grown on wheat roots and that the mutant and complemented strains performed as expected with respect to *pcsR2* gene expression. Transcript abundances of two genes in operon 2 (Pchl3084_4801, Pchl3084_4803) also were measured via qRT-PCR when bacteria were grown on wheat roots and planktonically (Figures 2C,D). Relative gene expression of Pchl3084_4801 and Pchl3084_4803 in 30–84WT were ∼10-fold higher when bacteria were grown on wheat roots as compared to in liquid culture, whereas the relative expression of both genes in operon 2 in the *pcsR2* mutant was not upregulated on roots, consistent with PcsR2 involvement in the upregulation of operon 2 on wheat roots. In both conditions, expression of Pchl3084_4801 and Pchl3084_4803 in 30–84ΔpcsR2(pGT2PcsR2) was higher than in 30–84WT, consistent with higher copy number of *pcsR2*, and similar to 30–84WT, relative gene expression of both genes in operon 2 in 30–84ΔpcsR2(pGT2PcsR2) were ∼6–10-fold higher when bacteria were grown on roots as compared to in liquid culture. These data are also consistent with PcsR2 involvement in the upregulation of operon 2 on wheat roots. The observation that the expression of Pchl3084_4801 and Pchl3084_4803 in the 30–84ΔpcsR2(pGT2PcsR2) was higher than in 30–84WT in AB + CAA, may indicate that the ligand is present at a low level in the absence of wheat roots.

### PcsR2 Needed for Root Macerate Utilization and Rhizosphere Colonization

We were curious whether *pcsR2* gene expression was responding specifically to root-derived substrates. The transcriptional reporter pGT2PpcsR2:gfp (containing *pcsR2* operon promoter fused to *gfp*) transformed into 30–84WT and 30–84ΔpcsR2 was used to quantify *pcsR2* expression when strains were grown planktonically in AB + G medium supplemented with or without AHLs, root macerate, or leaf macerate. In a preliminary experiment, we measured the expression of *pcsR2* over 24 h and observed that *pcsR2* expression plateaued at 12 h (Supplementary Figures 3A,B), so subsequent assays were performed at this time point. Addition of AHLs (extracted from 30 to 84ZN) had no effect on the expression of the reporter in either 30–84WT or 30–84ΔpcsR2 (data not shown). Moreover, the expression of pGT2PpcsR2:gfp or pGT2P4806:gfp (containing the operon 2 promoter fused to *gfp*) in QS mutant 30–84R (disrupted in *phzR*) was not significantly different from expression in 30–84WT, indicating neither *pcsR2* nor operon 2 is regulated by PhzR (data not shown).

**FIGURE 3:**
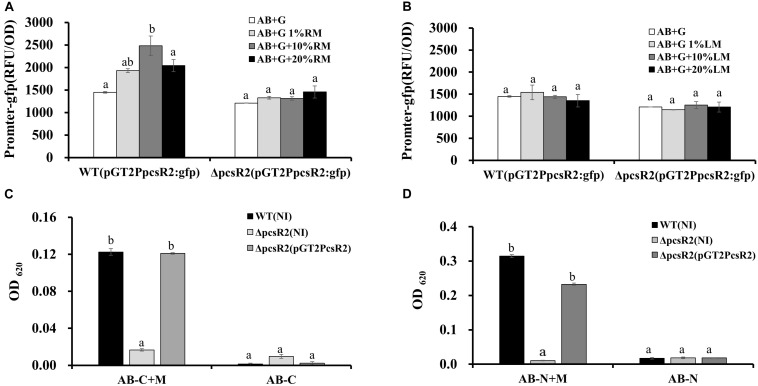
PcsR2 is involved in plant-microbe interactions. **(A,B)** Expression of the transcriptional *pcsR2* GFP reporter (pGT2PpcsR2:gfp) in 30–84WT and 30–84ΔpcsR2 when incubated in the presence of different concentrations of root or leaf macerate. 30–84WT and 30–84ΔpcsR2 carrying the reporter plasmid were incubated in 96 well plates for 12 h in AB medium with glucose (AB + G) without or with varying concentrations (1–20%) of root macerate (RM) **(A)** or Leaf macerate (LM) **(B)**. GFP fluorescence is expressed as relative fluorescence units (RFU) (fluorescence units standardized to population density). **(C,D)** Growth 30–84WT, 30–84ΔpcsR2, and 30–84ΔpcsR2(pGT2PcsR2) in AB medium without a carbon **(C)** or nitrogen **(D)** source and with 80% root macerate (AB-C + M or AB-N + M) or without it (AB-C or AB-N). Population density was measured spectrophotometrically (OD_620_) at 24 h of growth. NI means no insert control plasmid. Data are the means and standard errors of three replicates. Letters indicate whether treatments are significantly different (*P* < 0.05, ANOVA and Tukey HSD).

Relative GFP intensity (standardized to cell density) of pGT2PpcsR2:gfp in 30–84WT increased significantly with increasing concentration of root macerate up to 10%, but not with leaf macerate even at the highest concentration (Figures 3A,B). The relative GFP intensity of the reporter in the mutant was unchanged in both conditions, suggesting the mutant may be defective in signal uptake or signal sensing. These findings indicated *pcsR2* gene expression responds to unknown signals present in high enough concentration in wheat roots to be detected by the reporter, but not in leaves.

To test whether PcsR2 affects traits involved in utilization of root macerate as a carbon or nitrogen source, the three strains (30–84WT, 30–84ΔpcsR2, 30–84ΔpcsR2(pGT2PcsR2) were grown separately in modified AB medium supplemented with root macerate as the sole carbon (AB-C + M) or nitrogen (AB-N + M) source for 24 h. The modified AB without a carbon (AB-C) or nitrogen (AB-N) source was used as controls. None of the strains grew well without a supplemental carbon or nitrogen source. 30–84WT and ΔpcsR2(pGT2PcsR2) grew significantly better (as measured by OD_620_) than 30–84ΔpcsR2 in media supplemented with macerate (AB-C + M) and (AB-N + M). These results suggest PcsR2 effects traits involved in the uptake and/or utilization of carbon and nitrogen sources from wheat root exudates (Figures 3C,D).

To examine whether PcsR2 contributes to rhizosphere colonization and persistence, 30–84WT and 30–84ΔpcsR2 were inoculated separately into soil and after 4 days surface sterilized, pre-germinated wheat seedlings were planted. Bacteria were isolated from roots after 6 weeks and relative CFU (standardized to root dry weight) was calculated. Populations of 30–84WT on roots were significantly greater (∼10-fold) than populations of 30–84ΔpcsR2 (i.e., log_10_ (CFU/g) = 7.9 ± 0.2 vs. 6.8 ± 0.1, respectively).

### Loss of PcsR2 Affects Phenazine Production and Biofilm Traits

**FIGURE 4:**
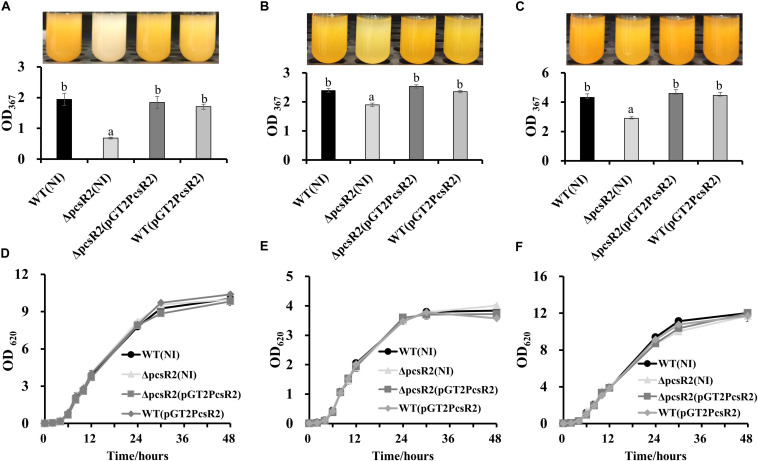
Phenazine production and growth of 30–84WT(NI), 30–84ΔpcsR2(NI), and 30–84ΔpcsR2(pGT2PcsR2) and 30–84WT(pGT2PcsR2) in different media. Strains were grown in **(A,D)** AB + CAA, **(B,E)** LB, and **(C,F)** PPMD at 28°C, for 48 h, with agitation. **(A–C)** Phenazines were quantified spectrophotometrically (OD_367_) and **(D–F)** growth curves were calculated spectrophotometrically (OD_620_). NI means no insert control plasmid. Data are the means and standard errors of at least three replicates; some error bars do not exceed the size of symbol. Letters indicate whether treatments are significantly different (*P* < 0.05, ANOVA and Tukey HSD).

An unexpected phenotype of the 30–84ΔpcsR2 was diminished phenazine production (Figure 4). When grown in AB + CAA (Figure 4A), total phenazines produced by 30–84WT and 30–84ΔpcsR2(pGT2PcsR2) were more than twofold greater than the amount of phenazines produced by the mutant (i.e., OD_367_ = 1.94 ± 0.20 and 1.84 ± 0.20 vs. 0.68 ± 0.02, respectively). However, when grown in media supporting higher phenazine production (e.g., LB or PPMD), the difference between wild type and the mutant in phenazine production was not as pronounced (Figures 4B,C). All strains, 30–84WT, 30–84ΔpcsR2, 30–84ΔpcsR2(pGT2PcsR2), 30–84WT(pGT2PcsR2) grew at a similar rate to each other in planktonic culture regardless of media type, indicating this reduction in phenazine production was not a function of population density (Figures 4D–F). These data indicate that a PcsR2 is required for wild type levels of phenazine production, that the defect is more pronounced in certain media, that the complementation of the defect restores phenazine production to wild type levels.

Given the effect of root macerate on *pcsR2* gene expression, we were curious whether root macerate would stimulate phenazine production. Phenazine production by 30–84WT was enhanced when this strain was grown in AB + G media with increasing root macerate supplementation, however phenazine production by 30–84ΔpcsR2 was almost unmeasurable and was unchanged by the addition of root macerate (Supplementary Figure 3C). These data are consistent with PcsR2 having an indirect role on phenazine production.

**FIGURE 5:**
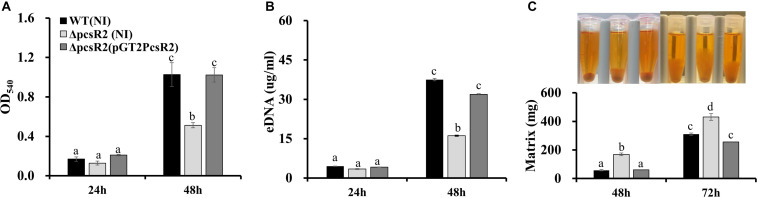
Biofilm traits of 30–84WT(NI), 30–84ΔpcsR2(NI), and 30–84ΔpcsR2(pGT2PcsR2). **(A)** Spectrophotometric quantification of surface-attached biofilms by the crystal violet staining method (at OD_540_) 24 and 48 h. **(B)** Quantification of extracellular (eDNA) when strains were grown in static culture after 24 and 48 h. **(C)** Image of biofilm matrix and mass of cells and hydrated matrix when strains grown in static culture measured by weight after 48 and 72 h. All figures use the same labeling for each strain. NI means no insert control plasmid. For all experiments, bacteria were grown in AB + CAA. Data are the means and standard errors of at least three replicates. Letters indicate whether treatments are significantly different (*P* < 0.05, ANOVA and Tukey HSD).

Because production of phenazines was shown previously to be essential for surface attached biofilm formation (Maddula et al., 2006) and to promote extracellular DNA (eDNA) release and extracellular matrix production (Wang et al., 2016), we hypothesized that PcsR2 may contribute to these phenotypes. When grown in AB + CAA, no differences were measured between 30 and 84WT, 30–84ΔpcsR2, and 30–84ΔpcsR2(pGT2PcsR2) in the number of surface-attached cells after 24 h growth in static culture or the amount of eDNA released into floating biofilm cultures. However, by 48 h the surface-attached populations observed for the 30–84WT and 30–84ΔpcsR2(pGT2PcsR2) increased dramatically and were significantly greater than observed for 30–84ΔpcsR2 (Figure 5A). eDNA production by 30–84WT and 30–84ΔpcsR2(pGT2PcsR2) was also significantly greater than by the mutant (Figure 5B). However, by 48 h, 30–84ΔpcsR2 produced significantly more extracellular matrix than 30–84WT or the 30–84ΔpcsR2(pGT2PcsR2) and the difference was dramatic after 72 h in static culture (Figure 5C). As expected, the extracellular matrix of all strains was completely disrupted by the addition of DNase1 (data not shown). Greater matrix production by the mutant was somewhat surprising given that it produced less eDNA, but eDNA was vital to extracellular matrix integrity. Taken together, these results indicate that the reduction in phenazine production associated with the loss of PcsR2 is likely responsible for most, but not all the changes in biofilm characteristics.

### Disruption of PcsR2 Reduced the Expression of *phzR/phzI* and Other Phenazine Regulators

**FIGURE 6:**
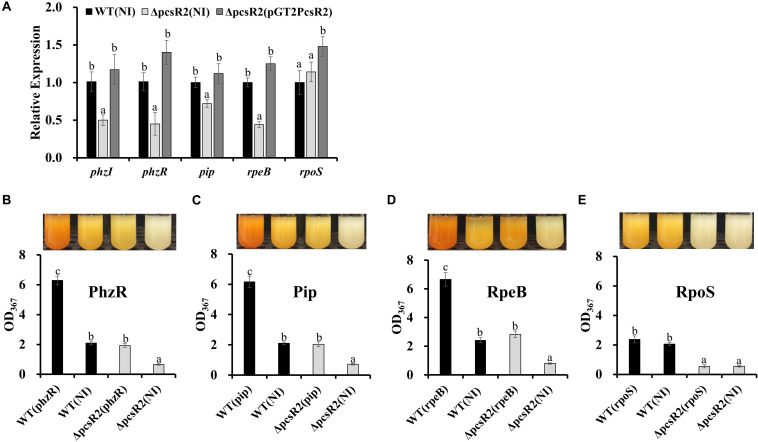
Gene expression of phenazine-regulators and in 30–84WT(NI), 30–84ΔpcsR2(NI), and 30–84ΔpcsR2(pGT2PcsR2) and phenazine production by 30–84WT, and 30–84ΔpcsR2 with and without additional copies of some phenazine regulatory proteins *in trans.* **(A)** Relative gene expression of some phenazine regulatory genes measured by qRT-qPCR (ΔΔCt method with *rpoD* as internal control) after 24 h in AB + CAA. Relative fold change of these genes is compared to 30–84WT expression levels (which are set to 1). **(B–E)** Phenazine production of strains with and without additional copies of *phzR*, *pip, rpeB*, or *rpoS*, via expression the following plasmids *in trans*: pGT2Ptac:phzR, pUCPip, pUCRpeB, pUCRpoS, respectively. NI means no insert control plasmid (pGT2 or pUCP20). Images above graphs are of cultures after 48 h growth in AB + CAA when phenazines were extracted and quantified spectrophotometrically (OD_367_). Data are the means and standard errors of five replicates. Letters indicate whether treatments are significantly different (*P* < 0.05, ANOVA and Tukey HSD).

Phenazine biosynthesis is regulated by a hierarchical network of regulatory genes. For example the quorum sensing genes PhzR/PhzI are regulated by the phenazine inducing protein Pip (not proline iminopeptidase), which in turn is regulated by the two-component signal transduction system RpeA/RpeB and under nutrient limited conditions the stationary sigma factor RpoS (Whistler and Pierson, 2003; Wang et al., 2012, 2013). In order to examine whether PcsR2 affects the expression of these regulators, we measured their transcript abundance in 30–84WT(NI), 30–84ΔpcsR2(NI), and 30–84ΔpcsR2(pGT2PcsR2) using qRT-PCR. The relative expression of regulators *phzI*, *phzR, pip*, and *rpeB* (Figure 6A) were significantly lower in 30–84ΔpcsR2(NI) compared to 30–84WT (NI) or 30–84ΔpcsR2(pGT2PcsR2). In contrast, the relative expression of *rpoS* did not differ significantly among the three strains. These results indicate that PcsR2 influences phenazine production via activity of RpeB, Pip, and PhzR/PhzI, but not RpoS.

To confirm the influence of PcsR2 on the RpeB/Pip/PhzR/PhzI regulatory network, extra copies of these regulatory genes under the control of a constitutive promoter were introduced into 30–84WT and 30–84ΔpcsR2. As expected, in 30–84WT constitutive expression of *phzR*, *pip*, or *rpeB*, but not *rpoS* resulted in a substantially increase in phenazine production. Significantly, in 30–84ΔpcsR2 constitutive expression of *phzR, pip, rpeB*, but not *rpoS* restored phenazine production to wild type levels (Figures 6B–E). Consistent with these results, supplementation of growth media with AHLs purified from 30 to 84ZN (defective in phenazine production) restored phenazine production in 30–84ΔpcsR2 to wild type levels (data not shown).

The promoter region for operon encoding the two component signal transduction system RpeA/RpeB at the top of the network lacks a *lux* box. These data suggest that PcsR2 modulates the production of phenazines via an as yet undetermined, indirect effect on the RpeA/RpeB regulatory network that includes Pip and PhzR/PhzI.

## Discussion

Plants and prokaryotes have coexisted and coevolved over millions of years in the rhizosphere. In this specific ecological niche, eukaryotes and prokaryotes communicate efficiently via chemically mediated interkingdom signaling, shaping their evolution and contemporary ecology (Hughes and Sperandio, 2008). Bacterial QS regulation via the production of AHLs, the prototypical QS signal, has been shown to be important for regulating key bacterial traits for bacteria-plant interactions, and the production of AHL mimics by plants highlights the importance of LuxR-based signal-response regulators as contributing to the recognition of the chemical language involved in interkingdom communication (Loh et al., 2002; Kan et al., 2017). LuxR solos comprise an interesting subgroup of LuxR regulators that may not necessarily respond to an AHL type molecule, and recent observations suggest that LuxR solos are widespread among Gram negative bacteria and may be more common than QS LuxRs in bacterial genomes (Subramoni and Venturi, 2009a; Hudaiberdiev et al., 2015). Thus LuxR solos are good candidates for being mediators of host-PGPR interactions by regulating traits involved in host recognition, host adaptation, or host growth-promoting activities in response to plant signals. In the present study, we focused on LuxR-type regulators in the genome of the well-characterized PGPR strain *P. chlororaphis* 30–84, which has a diversity of LuxRs, including QS LuxRs and typical and atypical solo LuxR-type regulators. We were particularly interested in plant-responsive gene regulation by any of the LuxR solos.

The twenty-five genes annotated as encoding LuxR-family transcriptional regulators in the *P. chlororaphis* 30–84 genome include the two previously described QS LuxRs (PhzR and CsaR) and twenty-three solo LuxR-type homologs. As is characteristic of LuxR-family proteins, they bear little amino acid sequence identity to one another and are annotated based on the presence of signal-binding and HTH DNA-binding domains. Only one of the LuxR solo homologs is predicted to have an autoinducer binding domain at the N-terminus (PcsR1, Table 2). PcsR1 has two substitutions of the nine highly conserved amino acids in typical QS LuxRs, suggesting it may not bind to AHLs produced by PhzI or CsaI [i.e., N-(3-OH-hexanoyl)- and N-(hexanoyl)-HSL or N-(butanoyl)-HSL, respectively, Khan et al., 2007], but may bind to exogenously produced AHLs (i.e., produced by other strains of bacteria) or other types of signals, although this remains to be tested. Interestingly, SmoR in *Stenotrophomonas maltophilia*, which bears the highest amino acid sequence identity to PcsR1 (53%) and has amino acid substitutions at the same two positions, is reported to respond to exogenously produced AHL, specifically oxo-C8-homoserine lactone (Martínez et al., 2015). The other twenty-two solo *P. chlororaphis* 30–84 LuxR-family homologs are atypical in that in place of the autoinducer binding domain, they have either PAS signal-sensing domains, REC/CheY signal receiver domains, or unidentified domains, indicating that they may respond to other types of exogenously-derived signals or stimuli. The N-terminal domains of one half of the atypical solo LuxR-family homologs are annotated as a REC/CheY family of response regulator domains. Response regulators that combine the REC/CheY domain with a DNA binding HTH domain are common in prokaryotic genomes. Typically, these response regulators are part of two-component signal transduction systems that respond to a variety of environmental stimuli or intracellular signals (Galperin, 2006). Interestingly, solo LuxR-family homologs Pchl3084_2371 and Pchl3084_3179 are annotated as having PAS-domains. PAS domains are a family of sensor protein domains involved in signal transduction and are found in all kingdoms of life. They have been shown to bind chemically diverse small-molecule metabolites and to serve as sensors of external stimuli (Henry and Crosson, 2011). For example, the gene downstream of Pchl3084_3179 is annotated as a chitinase. Thus, it may be reasonable to hypothesize that Pchl3084_3179 plays a sensory role in bacteria-fungi interactions by regulating the expression of this gene in direct response to fungal metabolites, although this has yet to be tested.

In the present study, we focused on the LuxR solo PcsR2 because its gene expression is upregulated ∼10-fold on roots. We showed that *pcsR2* expression is enhanced specifically in response to root-derived substrates as compared to leaf-derived substrates and does not respond to endogenous AHLs produced by wild type *P. chlororaphis* 30–84. Gene expression of the downstream operon also was highly upregulated on roots in wild type but not in the mutant, consistent with PcsR2 being involved in the regulation of this operon. As was shown via the contrasting behavior of the *pcsR2* mutant versus the wild type and complemented mutant, PcsR2s is involved in the metabolism of root-derived carbon and nitrogen, although it is unclear if this is due to PcsR2 effects on metabolite uptake or catabolism. The specificity of *pcsR2* transcriptional response is similar to the specificity of LuxR solos in some plant-beneficial *Pseudomonas* strains. For example, PipR in *Pseudomonas* sp. GM79, an endophyte of *Populus* responds specifically to metabolites in leaf but not root macerates. One particular component of leaf macerate, ethanolamine derivatives, was identified as specifically interacting with PipR (Schaefer et al., 2016; Coutinho et al., 2018). For PsoR in the biological control strains *P. protegnes* CHA0 and *P. protegens* Pf-5, the specificity is for plant signals derived from certain plant species, i.e., plant-produced molecules in rice and wheat but not in cucumber (Subramoni et al., 2011). In the present study, we show that *pcsR2* is upregulated in the presence of wheat root macerate but have yet to demonstrate a specific plant-derived signal interaction with PcsR2 on a protein level. However, it is also possible that a plant-derived signal is modified by *P. chlororaphis* 30–84 and that PcsR2 responds to this modified product. The observation that the expression of Pchl3084_4801 and Pchl3084_4803 in 30–84ΔpcsR2(pGT2PcsR2) was higher than in 30–84WT in AB + CAA, may indicate that the ligand is present at a low level in the absence of root exudates, that there is some induction of operon 2 by PcsR in the absence of ligand, or be an unknown consequence of the higher expression *pcsR2* in 30–84ΔpcsR2(pGT2PcsR2) than 30–84WT and warrants further study.

Our results suggest that PcsR2 is a member of a novel subfamily of LuxR-type transcriptional regulators found in *P. chlororaphis* that may be involved in the regulation of a conserved fatty acid desaturation locus. PcsR2 bears low amino acid identity with other well-characterized LuxR solos in plant-beneficial species such as LesR, PipR, and PsoR (∼20%) but high amino acid sequence identity (96–100%) with PcsR2 homologs only found in other sequenced strains of *P. chlororaphis* (Supplementary Table 2). Additionally, unlike the other plant responsive LuxR solos characterized to date, *pcsR2* is not associated with a proline iminopeptidase (*pip*) gene. It is part of a two-gene operon with an AMP-binding protein. Located downstream is a fatty acid desaturase containing operon (Supplementary Figure 1) and this operon is present and topologically organized in the same manner in all other sequenced *P. chlororaphis* strains with PcsR2 homologs (NCBI database, as of this date).

An interesting discovery was the involvement of PcsR2 in phenazine production. We observed that compared to wild type or the complemented mutant, phenazine production by the *pcsR2* mutant was significantly reduced even after cultures attained a high cell density and entered into stationary phase. Moreover, we found that phenazine production was enhanced in the wild type when grown in minimal media supplemented with root macerate, however, no such increase in phenazine was observed in the *pcsr2* mutant, consistent with and indirect role of PcsR2 in phenazine production. Compared to wild type and the complemented mutant, the formation of surface attached biofilm communities by the *pcsR2* mutant was also diminished. We hypothesize this biofilm phenotype is related to the reduced phenazine production by the mutant. This hypothesis is supported by previous reports showing that phenazine production is required for the formation of surface attached biofilm communities (Maddula et al., 2006). Phenazine production also was shown previously to contribute to eDNA release associated with autolysis, resulting in more extensive biofilm matrix production and viscosity (Das and Manefield, 2012; Das et al., 2015; Wang et al., 2016). Somewhat surprisingly, although the wild type and complemented mutant released large quantities of eDNA and produced extensive biofilm matrix, we found that the *pcsR2* mutant produced significantly more extracellular matrix, but significantly less eDNA. As expected, the extracellular matrix of the wild type, mutant and complemented mutant were completely disrupted by the addition of DNase1, indicating eDNA is required for matrix integrity, but the mutant must be contributing as yet unidentified component(s) to the matrix.

Our data suggest that PcsR2 affects phenazine production in *P. chlororaphis* 30–84 indirectly via the network of regulatory genes that control phenazine biosynthesis. Data presented in this study indicate that mutation of *pcsR2* results in a reduction in the expression of key phenazine regulatory genes *phzR, pip* (a gene encoding phenazine inducing protein, not proline iminopeptidase), and *rpeB*, and that phenazine production can be restored to wild-type levels by expressing any of these genes *in trans* in the mutant. These findings are in agreement with previous work that demonstrated phenazine biosysnthesis in *P. chlororaphis* 30–84 is regulated by the PhzR/PhzI QS system, which in turn is positively regulated by Pip (phenazine inducing protein) and the response regulator RpeB, part of the RpeA/RpeB two component signal transduction system (Wang et al., 2012). According to the model proposed in that study, the RpeA/RpeB two component system may function as a sensor of the metabolic or environmental stress condition of the cell, governing the production of secondary metabolites under stress conditions via the control of additional regulatory proteins. In that model, the membrane bound sensor kinase protein RpeA is proposed to control the level of active, phosphorylated RpeB, which in turn promotes the expression of *pip* and Pip in turn promotes the expression of *phzR* (Wang et al., 2012). Our data suggest PcsR2 acts upstream of RpeA/RpeB via an as yet undetermined mechanism. Although the function and role of the genes in the downstream operon have yet to be determined, we hypothesize that PcsR2 regulation of fatty acid desaturation may affect membrane fluidity. If so, this may affect the function of proteins bound in the outer membrane, including transport proteins or the receptors of two component systems such as RpeA that are likely to be involved in secondary metabolite production. Indeed, previous work using *Pseudomonas aeruginosa* has shown that mutations that disrupt the fatty acid profile of phospholipids alter membrane fluidity, resulting in altered production of QS signals, secondary metabolites, and traits associated survival (Baysse et al., 2005). Future work will focus on the function of the PcsR2-regulated genes that appear to be unique to *P. chlororaphis* and their roles in *P. chlororaphis* interactions with plants.

## Data Availability Statement

All datasets generated for this study are included in the article/Supplementary Material, further inquiries can be directed to the corresponding author.

## Author Contributions

EP and HP designed the whole experiments. HP carried out the experiments, performed the data analysis, and wrote the first draft. LP and EP contributed to the interpretation of results and revisions of the manuscript. EP provided project supervision and major contribution to the final draft. All authors read and approved the submitted version.

## Conflict of Interest

The authors declare that the research was conducted in the absence of any commercial or financial relationships that could be construed as a potential conflict of interest.

## Footnotes

1 https://www.ncbi.nlm.nih.gov/

2 https://www.ncbi.nlm.nih.gov/

3 http://www.pseudomonas.com/

4 https://www.ncbi.nlm.nih.gov/Structure/cdd/wrpsb.cgi

5 https://pfam.xfam.org/

6 http://www.ebi.ac.uk/Tools/msa/clustalo/

7 https://embnet.vital-it.ch/software/BOX_form.html

## References

[B1] Ahlgren N. A. Harwood C. S. Schaefer A. L. Giraud E. Greenberg E. P. (2011). Aryl-homoserine lactone quorum sensing in stem-nodulating photosynthetic bradyrhizobia. *Proc. Natl. Acad. Sci. U.S.A.* 108 7183–7188. 10.1073/pnas.1103821108 21471459 PMC3084126

[B2] Baysse C. Cullinane M. Dénervaud V. Burrowes E. Dow J. M. Morrissey J. P. (2005). Modulation of quorum sensing in *Pseudomonas aeruginosa* through alteration of membrane properties. *Microbiology* 151 2529–2542. 10.1099/mic.0.28185-0 16079332

[B3] Brachmann A. O. Brameyer S. Kresovic D. Hitkova I. Kopp Y. Manske C. (2013). Pyrones as bacterial signaling molecules. *Nat. Chem. Biol.* 9 573–578. 10.1038/nchembio.1295 23851573

[B4] Brameyer S. Bode H. B. Heermann R. (2015a). Languages and dialects: bacterial communication beyond homoserine lactones. *Trends Microbiol.* 23 521–523. 10.1016/j.tim.2015.07.002 26231578

[B5] Brameyer S. Kresovic D. Bode H. B. Heermann R. (2014). LuxR solos in Photorhabdus species. *Front. Cell. Infect. Microbiol.* 4:166. 10.3389/fcimb.2014.00166 25478328 PMC4235431

[B6] Brameyer S. Kresovic D. Bode H. B. Heermann R. (2015b). Dialkylresorcinols as bacterial signaling molecules. *Proc. Natl. Acad. Sci. U.S.A.* 112 572–577. 10.1073/pnas.1417685112 25550519 PMC4299209

[B7] Chatnaparat T. Prathuangwong S. Ionescu M. Lindow S. E. (2012). XagR, a LuxR homolog, contributes to the virulence of *Xanthomonas axonopodis* pv. *glycines* to soybean. *Mol. Plant Microbe Interact.* 25 1104–1117. 10.1094/mpmi-01-12-0008-r 22746827

[B8] Chiang P. Burrows L. L. (2003). Biofilm formation by hyperpiliated mutants of *Pseudomonas aeruginosa*. *J. Bacteriol.* 185 2374–2378. 10.1128/jb.185.7.2374-2378.2003 12644510 PMC151504

[B9] Corral-Lugo A. Daddaoua A. Ortega A. Espinosa-Urgel M. Krell T. (2016). Rosmarinic acid is a homoserine lactone mimic produced by plants that activates a bacterial quorum-sensing regulator. *Sci. Signal.* 9:ra1. 10.1126/scisignal.aaa8271 26732761

[B10] Coutinho B. G. Mevers E. Schaefer A. L. Pelletier D. A. Harwood C. S. Clardy J. (2018). A plant-responsive bacterial-signaling system senses an ethanolamine derivative. *Proc. Natl. Acad. Sci. U.S.A.* 115 9785–9790. 10.1073/pnas.1809611115 30190434 PMC6166808

[B11] Das T. Kutty S. K. Tavallaie R. Ibugo A. I. Panchompoo J. Sehar S. (2015). Phenazine virulence factor binding to extracellular DNA is important for *Pseudomonas aeruginosa* biofilm formation. *Sci. Rep.* 5:8398. 10.1038/srep08398 25669133 PMC4323658

[B12] Das T. Manefield M. (2012). Pyocyanin promotes extracellular DNA release in *Pseudomonas aeruginosa*. *PLoS One* 7:e46718. 10.1371/journal.pone.0046718 23056420 PMC3466280

[B13] de Bruijn I. Raaijmakers J. M. (2009). Diversity and functional analysis of LuxR-type transcriptional regulators of cyclic lipopeptide biosynthesis in *Pseudomonas fluorescens*. *Appl. Environ. Microbiol.* 75 4753–4761. 10.1128/AEM.00575-09 19447950 PMC2708414

[B14] Dorosky R. J. Pierson L. S. Pierson E. A. (2018). Produces multiple R-tailocin particles that broaden the killing spectrum and contribute to persistence in rhizosphere communities. *Appl. Environ. Microbiol.* 84 e01230–18. 10.1128/aem.01230-18 30030224 PMC6121977

[B15] Engebrecht J. Nealson K. Silverman M. (1983). Bacterial bioluminescence: isolation and genetic analysis of functions from *Vibrio fischeri*. *Cell* 32 773–781. 10.1016/0092-8674(83)90063-6 6831560

[B16] Ferluga S. Bigirimana J. Hofte M. Venturi V. (2007). A LuxR homologue of *Xanthomonas oryzae* pv. *oryzae* is required for optimal rice virulence. *Mol. Plant Pathol.* 8 529–538. 10.1111/j.1364-3703.2007.00415.x 20507519

[B17] Ferluga S. Venturi V. (2009). OryR Is a LuxR-family protein involved in interkingdom signaling between pathogenic *Xanthomonas oryzae* pv. *oryzae* and Rice. *J. Bacteriol.* 191 890–897. 10.1128/jb.01507-08 19028884 PMC2632065

[B18] Fuqua C. (2006). The QscR quorum-sensing regulon of *Pseudomonas aeruginosa*: an orphan claims its identity. *J. Bacteriol.* 188 3169–3171. 10.1128/jb.188.9.3169-3171.2006 16621807 PMC1447470

[B19] Fuqua C. Greenberg E. P. (2002). Listening in on bacteria: acyl-homoserine lactone signalling. *Nat. Rev. Mol. Cell Biol.* 3 685–695. 10.1038/nrm907 12209128

[B20] Fuqua W. C. Winans S. C. (1994). A LuxR-LuxI type regulatory system activates *Agrobacterium* Ti plasmid conjugal transfer in the presence of a plant tumor metabolite. *J. Bacteriol.* 176 2796–2806. 10.1128/jb.176.10.2796-2806.1994 8188582 PMC205432

[B21] Fuqua W. C. Winans S. C. Greenberg E. P. (1994). Quorum sensing in bacteria: the LuxR-LuxI family of cell density-responsive transcriptional regulators. *J. Bacteriol.* 176 269–275. 10.1128/jb.176.2.269-275 8288518 PMC205046

[B22] Galperin M. Y. (2006). Structural classification of bacterial response regulators: diversity of output domains and domain combinations. *J. Bacteriol.* 188 4169–4182. 10.1128/jb.01887-05 16740923 PMC1482966

[B23] Gonzalez J. F. Myers M. P. Venturi V. (2013). The inter-kingdom solo OryR regulator of *Xanthomonas oryzae* is important for motility. *Mol. Plant Pathol.* 14 211–221. 10.1111/j.1364-3703.2012.00843.x 23083431 PMC6638885

[B24] Gonzalez J. F. Venturi V. (2013). A novel widespread interkingdom signaling circuit. *Trends Plant Sci.* 18 167–174. 10.1016/j.tplants.2012.09.007 23089307

[B25] Grindley N. Joyce C. (1981). Analysis of the structure and function of the kanamycin-resistance transposon Tn903. *Cold Spring Harb. Symp. Quant. Biol.* 45(Pt 1) 125–133. 10.1101/sqb.1981.045.01.021 6271455

[B26] Henry J. T. Crosson S. (2011). Ligand-binding PAS domains in a genomic, cellular, and structural context. *Annu. Rev. Microbiol.* 65 261–286. 10.1146/annurev-micro-121809-151631 21663441 PMC3298442

[B27] Hmelo L. R. Borlee B. R. Almblad H. Love M. E. Randall T. E. Tseng B. S. (2015). Precision-engineering the *Pseudomonas aeruginosa* genome with two-step allelic exchange. *Nature Protoc.* 10:1820. 10.1038/nprot.2015.115 26492139 PMC4862005

[B28] Hoang T. T. Karkhoff-Schweizer R. R. Kutchma A. J. Schweizer H. P. (1998). A broad-host-range Flp-FRT recombination system for site-specific excision of chromosomally-located DNA sequences: application for isolation of unmarked *Pseudomonas aeruginosa* mutants. *Gene* 212 77–86. 10.1016/S0378-1119(98)00130-9 9661666

[B29] Hudaiberdiev S. Choudhary K. S. Alvarez R. V. Gelencser Z. Ligeti B. Lamba D. (2015). Census of solo LuxR genes in prokaryotic genomes. *Front. Cell. Infect. Microbiol.* 5:20. 10.3389/fcimb.2015.00020 25815274 PMC4357305

[B30] Hughes D. T. Sperandio V. (2008). Inter-kingdom signalling: communication between bacteria and their hosts. *Nature Rev. Microbiol.* 6 111–120. 10.1038/nrmicro1836 18197168 PMC2667375

[B31] Kan J. H. Fang R. X. Jia Y. T. (2017). Interkingdom signaling in plant-microbe interactions. *Sci. China Life Sci.* 60, 785–796. 10.1007/s11427-017-9092-3 28755299

[B32] Khan S. R. Herman J. Krank J. Serkova N. J. Churchill M. E. A. Suga H. (2007). N-(3-hydroxyhexanoyl)-l-homoserine lactone is the biologically relevant quormone that regulates the phz operon of *Pseudomonas chlororaphis* strain 30-84. *Appl. Environ. Microbiol.* 73 7443–7455. 10.1128/AEM.01354-07 17921283 PMC2168216

[B33] Kumar S. Stecher G. Tamura K. (2016). MEGA7: molecular evolutionary genetics analysis version 7.0 for bigger datasets. *Mol. Biol. Evol.* 33, 1870–1874. 10.1093/molbev/msw054 27004904 PMC8210823

[B34] Loh J. Pierson E. A. Pierson L. S. Stacey G. Chatterjee A. (2002). Quorum sensing in plant-associated bacteria. *Curr. Opin. Plant Biol.* 5 285–290. 10.1016/S1369-5266(02)00274-1 12179960

[B35] Loper J. E. Hassan K. A. Mavrodi D. V. Davis E. W. II Lim C. K. Shaffer B. T. (2012). Comparative genomics of plant-associated *Pseudomonas* spp.: insights into diversity and inheritance of traits involved in multitrophic interactions. *PLoS Genet.* 8:e1002784. 10.1371/journal.pgen.1002784 22792073 PMC3390384

[B36] Maddula V. Zhang Z. Pierson E. A. Pierson L. S. (2006). Quorum sensing and phenazines are involved in biofilm formation by *Pseudomonas chlororaphis* (*aureofaciens*) strain 30-84. *Microbial Ecol.* 52 289–301. 10.1007/s00248-006-9064-6 16897305

[B37] Mahmoudi T. R. Yu J. M. Liu S. Pierson L. S. III Pierson E. A. (2019). Drought-stress tolerance in wheat seedlings conferred by phenazine-producing rhizobacteria. *Front. Microbiol.* 10:1590. 10.3389/fmicb.2019.01590 31354678 PMC6636665

[B38] Martínez P. Huedo P. Martinez-Servat S. Planell R. Ferrer-Navarro M. Daura X. (2015). *Stenotrophomonas maltophilia* responds to exogenous AHL signals through the LuxR solo SmoR (Smlt1839). *Front. Cell. Infect. Microbiol.* 5:41. 10.3389/fcimb.2015.00041 26029670 PMC4432800

[B39] Mazzola M. Cook R. J. Thomashow L. S. Weller D. M. Pierson L. S. (1992). Contribution of phenazine antibiotic biosynthesis to the ecological competence of fluorescent pseudomonads in soil habitats. *Appl. Environ. Microbiol.* 58 2616–2624. 10.1128/AEM.58.8.2616-2624.1992 1514808 PMC195829

[B40] Miller W. G. Leveau J. H. J. Lindow S. E. (2000). Improved *gfp* and *inaz* broad-host-range promoter-probe vectors. *Mol. Plant Microbe Interact.* 13 1243–1250. 10.1094/mpmi.2000.13.11.1243 11059491

[B41] Mukherjee S. Bassler B. L. (2019). Bacterial quorum sensing in complex and dynamically changing environments. *Nat. Rev. Microbiol.* 17 371–382. 10.1038/s41579-019-0186-5 30944413 PMC6615036

[B42] Nealson K. H. Hastings J. W. (1979). Bacterial bioluminescence: its control and ecological significance. *Microbiol. Rev.* 43 496–518. 10.1128/mmbr.43.4.496-518.1979 396467 PMC281490

[B43] Papenfort K. Silpe J. E. Schramma K. R. Cong J.-P. Seyedsayamdost M. R. Bassler B. L. (2017). A *Vibrio cholerae* autoinducer–receptor pair that controls biofilm formation. *Nat. Chem. Biol.* 13 551–557. 10.1038/nchembio.2336 28319101 PMC5391282

[B44] Patankar A. V. González J. E. (2009). An orphan LuxR homolog of *Sinorhizobium meliloti* affects stress adaptation and competition for nodulation. *Appl. Environ. Microbiol.* 75 946–955. 10.1128/aem.01692-08 19088317 PMC2643561

[B45] Patel H. K. Ferrante P. Covaceuszach S. Lamba D. Scortichini M. Venturi V. (2014). The Kiwifruit emerging pathogen *Pseudomonas syringae* pv. *actinidiae* does not produce AHLs but possesses three LuxR solos. *PLoS One* 9:e87862. 10.1371/journal.pone.0087862 24498215 PMC3909224

[B46] Pierson E. A. Wang D. Pierson L. S. III (2013). “Roles and regulation of phenazines in the biological control strain *Pseudomonas chlororaphis* 30-84,” in *Microbial Phenazines*, eds Chincholkar S. Thomashow L. (Berlin: Springer), 141–162. 10.1007/978-3-642-40573-0_7

[B47] Pierson L. S. Keppenne V. D. Wood D. W. (1994). Phenazine antibiotic biosynthesis in *Pseudomonas aureofaciens* 30-84 is regulated by PhzR in response to cell density. *J. Bacteriol.* 176 3966–3974. 10.1128/jb.176.13.3966-3974.1994 8021179 PMC205594

[B48] Pierson L. S. Pierson E. A. (1996). Phenazine antibiotic production in *Pseudomonas aureofaciens*: role in rhizosphere ecology and pathogen suppression. *FEMS Microbiol. Lett.* 136 101–108. 10.1111/j.1574-6968.1996.tb08034.x

[B49] Pierson L. S. Pierson E. A. (2007). Roles of diffusible signals in communication among plant-associated bacteria. *Phytopathology* 97 227–232. 10.1094/phyto-97-2-0227 18944379

[B50] Pierson L. S. Pierson E. A. (2010). Metabolism and function of phenazines in bacteria: impacts on the behavior of bacteria in the environment and biotechnological processes. *Appl. Microbiol. Biotechnol.* 86 1659–1670. 10.1007/s00253-010-2509-3 20352425 PMC2858273

[B51] Pierson L. S. Thomashow L. S. (1992). Cloning and heterologous expression of the phenazine biosynthetic locus from *Pseudomonas-aureofaciens* 30-84. *Mol. Plant Microbe Interact.* 5 330–339. 10.1094/mpmi-5-330 1325219

[B52] Pierson L. S. Wood D. W. Pierson E. A. (1998). Homoserine lactone-mediated gene regulation in plant-associated bacteria. *Annu. Rev. Phytopathol.* 36 207–225. 10.1146/annurev.phyto.36.1.207 15012498

[B53] Piper K. R. von Bodman S. B. Farrand S. K. (1993). Conjugation factor of *Agrobacterium tumefaciens* regulates Ti plasmid transfer by autoinduction. *Nature* 362 448–450. 10.1038/362448a0 8464476

[B54] Qian G. L. Xu F. F. Venturi V. Du L. C. Liu F. Q. (2014). Roles of a solo LuxR in the biological control agent *Lysobacter enzymogenes* Strain OH11. *Phytopathology* 104 224–231. 10.1094/phyto-07-13-0188-r 24111575 PMC4161204

[B55] Recinos D. A. Sekedat M. D. Hernandez A. Cohen T. S. Sakhtah H. Prince A. S. (2012). Redundant phenazine operons in *Pseudomonas aeruginosa* exhibit environment-dependent expression and differential roles in pathogenicity. *Proc. Natl. Acad. Sci. U.S.A.* 109 19420–19425. 10.1073/pnas.1213901109 23129634 PMC3511076

[B56] Reddy S. K. Liu S. Rudd J. C. Xue Q. Payton P. Finlayson S. A. (2014). Physiology and transcriptomics of water-deficit stress responses in wheat cultivars TAM 111 and TAM 112. *J. Plant Physiol.* 171 1289–1298. 10.1016/j.jplph.2014.05.005 25014264

[B57] Rudd J. Devkota R. Baker J. Peterson G. Lazar M. Bean B. (2014). ‘TAM 112’ wheat, resistant to greenbug and wheat curl mite and adapted to the dryland production system in the southern high plains. *J. Plant Registrations* 8 291 10.3198/jpr2014.03.0016crc

[B58] Schaefer A. L. Greenberg E. P. Oliver C. M. Oda Y. Huang J. J. Bittan-Banin G. (2008). A new class of homoserine lactone quorum-sensing signals. *Nature* 454 595–599. 10.1038/nature07088 18563084

[B59] Schaefer A. L. Oda Y. Coutinho B. G. Pelletier D. A. Weiburg J. Venturi V. (2016). A LuxR homolog in a cottonwood tree endophyte that activates gene expression in response to a plant signal or specific peptides. *mBio* 7:e01101-16. 10.1128/mBio.01101-16 27486195 PMC4981722

[B60] Schuster M. Greenberg E. P. (2006). A network of networks: quorum-sensing gene regulation in *Pseudomonas aeruginosa*. *Int. J. Med. Microbiol.* 296 73–81. 10.1016/j.ijmm.2006.01.036 16476569

[B61] Subramoni S. Gonzalez J. F. Johnson A. Péchy-Tarr M. Rochat L. Paulsen I. (2011). Bacterial subfamily of LuxR regulators that respond to plant compounds. *Appl. Environ. Microbiol.* 77 4579–4588. 10.1128/aem.00183-11 21531826 PMC3127701

[B62] Subramoni S. Venturi V. (2009a). LuxR-family ‘solos’: bachelor sensors/regulators of signalling molecules. *Microbiology* 155 1377–1385. 10.1099/mic.0.026849-0 19383698

[B63] Subramoni S. Venturi V. (2009b). PpoR is a conserved unpaired LuxR solo of *Pseudomonas putida* which binds N-acyl homoserine lactones. *BMC Microbiol.* 9:125. 10.1186/1471-2180-9-125 19534812 PMC2703642

[B64] Teplitski M. Robinson J. B. Bauer W. D. (2000). Plants secrete substances that mimic bacterial N-acyl homoserine lactone signal activities and affect population density-dependent behaviors in associated bacteria. *Mol. Plant Microbe Interact.* 13 637–648. 10.1094/mpmi.2000.13.6.637 10830263

[B65] Venturi V. Fuqua C. (2013). Chemical signaling between plants and plant pathogenic bacteria. *Annu. Rev. Phytopathol.* 51 17–37. 10.1146/annurev-phyto-082712-102239 23915131

[B66] Wang D. Yu J. M. Dorosky R. J. Pierson L. S. III Pierson E. A. (2016). The phenazine 2-hydroxy-phenazine-1-carboxylic acid promotes extracellular dna release and has broad transcriptomic consequences in *Pseudomonas chlororaphis* 30–84. *PLoS One* 11:e0148003. 10.1371/journal.pone.0148003 26812402 PMC4727817

[B67] Wang D. P. Lee S. H. Seeve C. Yu J. M. Pierson L. S. Pierson E. A. (2013). Roles of the Gac-Rsm pathway in the regulation of phenazine biosynthesis in *Pseudomonas chlororaphis* 30-84. *MicrobiologyOpen* 2 505–524. 10.1002/mbo3.90 23606419 PMC3684763

[B68] Wang D. P. Yu J. M. Pierson L. S. Pierson E. A. (2012). Differential regulation of phenazine biosynthesis by RpeA and RpeB in *Pseudomonas chlororaphis* 30-84. *Microbiology* 158 1745–1757. 10.1099/mic.0.059352-0 22539162

[B69] Wang N. Lu S.-E. Records A. R. Gross D. C. (2006). Characterization of the transcriptional activators SalA and SyrF, which are required for syringomycin and syringopeptin production by *Pseudomonas syringae* pv. *syringae*. *J. Bacteriol.* 188 3290–3298. 10.1128/jb.188.9.3290-3298.2006 16621822 PMC1447436

[B70] Wang Y. Kern S. E. Newman D. K. (2010). Endogenous phenazine antibiotics promote anaerobic survival of *Pseudomonas aeruginosa* via extracellular electron transfer. *J. Bacteriol.* 192 365–369. 10.1128/jb.01188-09 19880596 PMC2798253

[B71] Wang Y. Wilks J. C. Danhorn T. Ramos I. Croal L. Newman D. K. (2011). Phenazine-1-carboxylic acid promotes bacterial biofilm development via ferrous iron acquisition. *J. Bacteriol.* 193 3606–3617. 10.1128/jb.00396-11 21602354 PMC3133341

[B72] Waters C. M. Bassler B. L. (2005). Quorum Sensing: cell-to-cell communication in bacteria. *Annu. Rev. Cell Dev. Biol.* 21 319–346. 10.1146/annurev.cellbio.21.012704.131001 16212498

[B73] Whistler C. A. Pierson L. S. III (2003). Repression of phenazine antibiotic production in *Pseudomonas aureofaciens* strain 30-84 by RpeA. *J. Bacteriol.* 185 3718–3725. 10.1128/jb.185.13.3718-3725.2003 12813064 PMC161564

[B74] Wood D. W. Gong F. Daykin M. M. Williams P. Pierson L. S. (1997). N-acyl-homoserine lactone-mediated regulation of phenazine gene expression by *Pseudomonas aureofaciens* 30-84 in the wheat rhizosphere. *J. Bacteriol.* 179 7663–7670. 10.1128/jb.179.24.7663-7670.1997 9401023 PMC179727

[B75] Wood D. W. Pierson L. S. (1996). The *phzI* gene of *Pseudomonas aureofaciens* 30–84 is responsible for the production of a diffusible signal required for phenazine antibiotic production. *Gene* 168 49–53. 10.1016/0378-1119(95)00754-7 8626064

[B76] Xu H. Y. Zhao Y. C. Qian G. L. Liu F. Q. (2015). XocR, a LuxR solo required for virulence in *Xanthomonas oryzae* pv. *oryzicola*. *Front. Cell. Infect. Microbiol.* 5:37. 10.3389/fcimb.2015.00037 25932456 PMC4399327

[B77] Yu J. M. Wang D. P. Pierson L. S. Pierson E. A. (2017). Disruption of MiaA provides insights into the regulation of phenazine biosynthesis under suboptimal growth conditions in Pseudomonas chlororaphis 30-84. *Microbiology (Reading)* 163, 94–108. 10.1099/mic.0.000409 27926818

[B78] Yu J. M. Wang D. P. Ries T. R. Pierson L. S. Pierson E. A. (2018). An upstream sequence modulates phenazine production at the level of transcription and translation in the biological control strain *Pseudomonas chlororaphis* 30-84. *PLoS One* 13:e0193063. 10.1371/journal.pone.0193063 29451920 PMC5815613

[B79] Zhang L. L. Jia Y. T. Wang L. Fang R. X. (2007). A proline iminopeptidase gene upregulated in planta by a LuxR homologue is essential for pathogenicity of *Xanthomonas campestris* pv. *campestris*. *Mol. Microbiol.* 65 121–136. 10.1111/j.1365-2958.2007.05775.x 17581124

[B80] Zhang Z. G. Pierson L. S. (2001). A second quorum-sensing system regulates cell surface properties but not phenazine antibiotic production in *Pseudomonas aureofaciens*. *Appl. Environ. Microbiol.* 67 4305–4315. 10.1128/aem.67.9.4305-4315.2001 11526037 PMC93161

