# Supplementary material for: PcsR2 Is a LuxR-Type Regulator That Is Upregulated on Wheat Roots and Is Unique to Pseudomonas chlororaphis
Source: Front Microbiol. 2020 Nov 10;11:560124. doi: 10.3389/fmicb.2020.560124 (PMC7683790; doi:10.3389/fmicb.2020.560124)
Supplement: Supplementary file 1 [file Data_Sheet_1.pdf]

Supplementary Material

Pan, H., Pierson, L.S., Pierson, E.A. (2020). PcsR2 is a LuxR-type regulator that is upregulated on wheat roots and is unique to *Pseudomonas chlororaphis*. *Frontiers in Microbiology*

Content:

Supplemental Figure 1 | The genomic region surrounding the LuxR homolog *pcsR2* (Pchl3084_4807) in *P. chlororaphis* 30-84

Supplemental Figure 2 | Molecular phylogenetic analysis of PcsR2 in *Pseudomonas*

Supplemental Figure 3 |The growth curve and promoter activity of *pscR2* of 30-84WT and 30-84ΔpcsR2 and phenazine production of 30-84WT and 30-84ΔpcsR2 with and without root macerate in AB+G.

Supplemental Table 1 | Primers used for gene cloning and qRT-PCR

Supplemental Table 2 | Amino acid identity of PcsR2 with other LuxR family regulators

1 Supplementary Figure 1

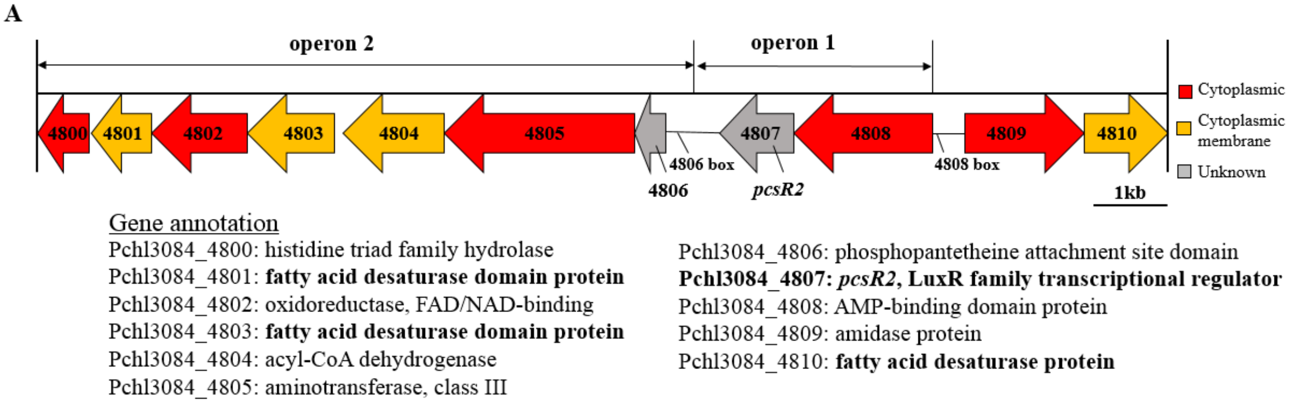

B

lux box	ACCTGTAG**GATC**GTACAGGT
phz box	CACTACAA**GATC**TGGTAGTT
4806 box	CAAGCGTT**GATC**CGCTGGTT
4808 box	CAGAGGAT**GATC**AAGCGTAA

Supplemental Figure 1 | The genomic region surrounding the LuxR homolog *pcsR2* (Pchl3084_4807) in *P. chlororaphis* 30-84**.** **(A)** The schematic was adapted from the *Pseudomonas* Genome Database (http://www.pseudomonas.com/). This region from Pchl3084_4800 to Pchl3084_4810 spans 14,475bp in total and contains eleven genes in three operons, among which are three fatty acid desaturase domain proteins. Arrows indicate orientation of transcription. Red indicates proteins predicted to be in the cytoplasm, orange indicates proteins predicted to be in the cytoplasmic membrane, the cellular location of products in gray is unknown. The location of putative lux box sequences in the promoter regions of Pchl3084_4808 (operon containing *pcsR2*) and Pchl3084_4806 (operon 2) are indicated. **(B)** Sequences of the predicted Pchl3084_4808 and Pchl3084_4806 *lux* boxes are provided in comparison to the *lux* box from *Vibrio fischeri* and the *phz* box from *P. chlororaphis* 30-84. The bases indicated in bold are shared among all sequences and the underlined bases are shared among at least two sequences.

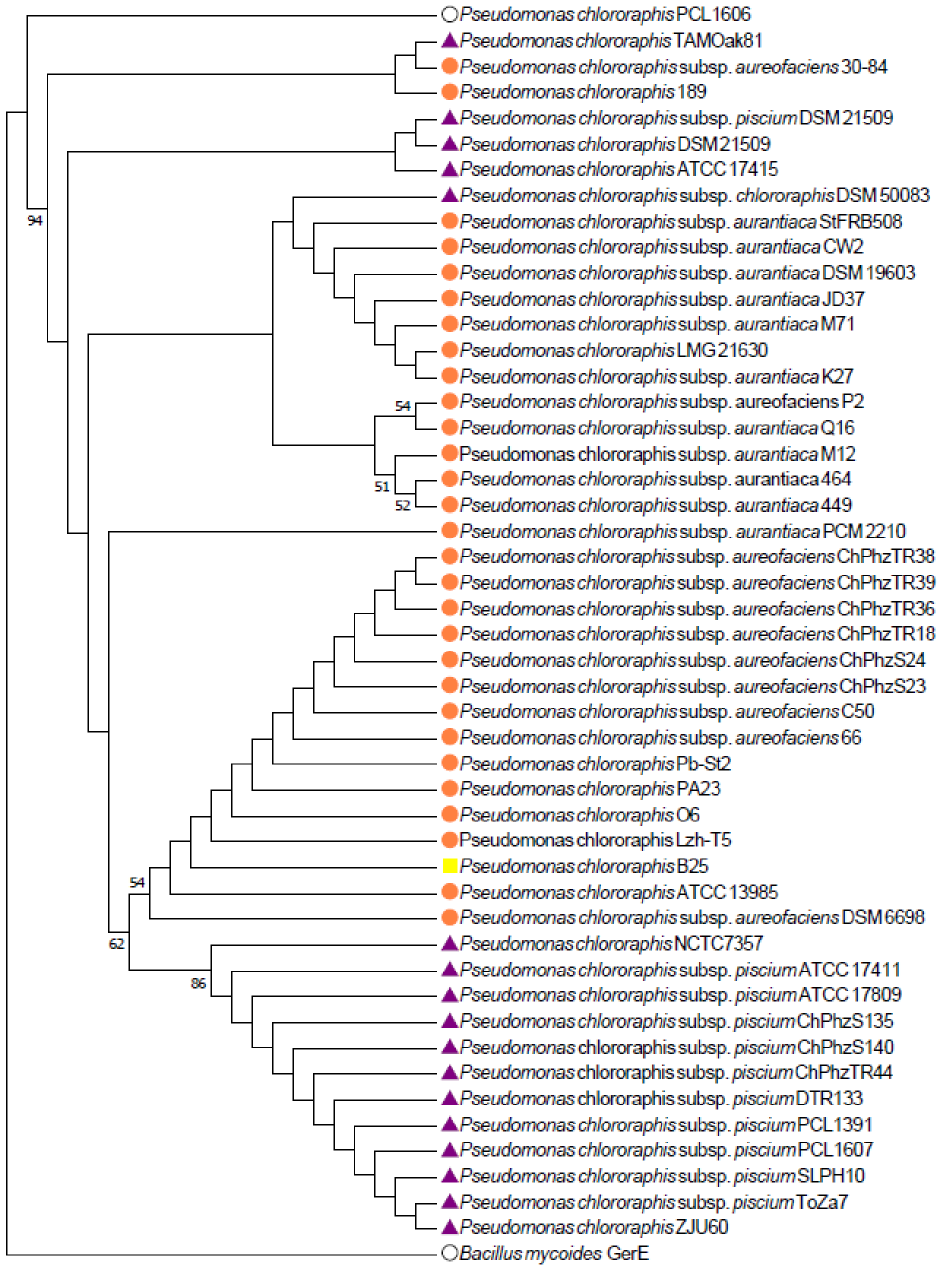

Supplemental Figure 2 | Molecular phylogenetic analysis of PcsR2 in *Pseudomonas*. The maximum likelihood (ML) phylogenetic tree was constructed from multiple-sequence alignments of PcsR2 homologs in 48 fully sequenced strains of *P. chlororaphis* with MEGA7 using MUSCLE (MUltiple Sequence Comparison by Log- Expectation) and bootstrap analysis with 1000 replicates. Values greater than 50 are indicated at the nodes. The closed circles (orange) indicate strains producing 2-hydroxypenzaine (2-OH-PHZ) and/or 2-hydroxypenazine-1-carboxylic acid (2-OH-PCA). The closed triangles (purple) indicate strains producing only pheanzine-1-carboxamide (PCN). The closed rectangles (yellow) indicate strains producing pheanzine-1-carboxylic acid (PCA). The open circle indicates strains that are not known to produce phenazines. GerE (a LuxR homlog) from *Bacillus mycoides* is used as a outgroup protein. Phenazine production was inferred from bioinformatic analysis by (Biessy et al., 2019).

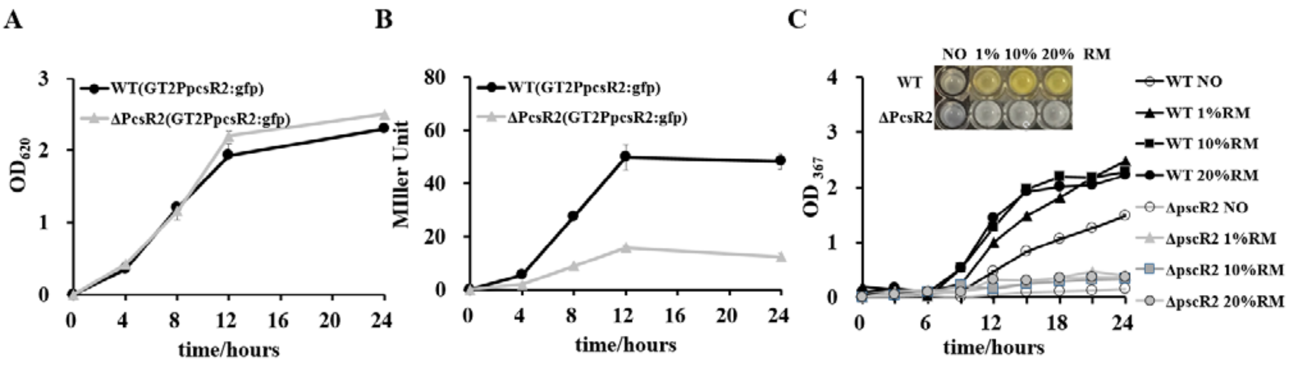

Supplemental Figure 3: The growth curve and promoter activity of *pscR2* of 30-84WT and 30-84ΔpcsR2 in AB+G and phenazine production of 30-84WT and 30-84ΔpcsR2 with and without root macerate. **(A)** Growth curve of 30-84WT and 30-84ΔpcsR2 with the reporter(pGT2PpcsR2:gfp) in AB+G media measured spectrophotometrically (OD_{620}) **(B**) The promoter activity of *pcsR2* in 30-84WT and 30-84ΔpcsR2 with reporter in AB+G media over 24 h based on ß-galactosidase activity (Miller Units); **(C)** Phenazine production of 30-84WT and 30-84ΔpcsR2 with and without root macerate (RM) measured spectrophotometrically (OD_{367}). The inset picture was taken at 24 h. In all figures data means ± SE, and in some cases SE bars do not exceed the markers.

Supplemental Table 1 | Oligonucleotides used for gene cloning and qPCR

Oligonucleotide	Sequence (5'-3')
4807KO-1	CGgaattcCCTGATTATCGTCAATGGCG (*Eco*RI)
4807KO-2	TCACTCGTCctgcagACTGGTCATGCTTCCACGA (*Pst*I)
4807KO-3	ATGACCAGTctgcagGACGAGTGAGTGCTGCCTG (*Pst*I)
4807KO-4	GGaagcttCTTTGGCTTTTGATCTACCCG (*Hin*dIII)
4807Check1	ACGGCGAGCTGTTCGTCA
4807Check2	CTACAGGGTTTAGCGCCGA
4807qPCR-R	TGTTGTCCATCCTGTCCAGC
4807-F-BamHI	CGGggatccCAGGTCGTGGAAGCAT (*Bam*HI)
4807-R-HindIII	CCCaagcttTCACTCGTCGCAGAACAA (*Hin*dIII)
4808pr-EcoRI-F	CGgaattcTGCGCACGCTGCTGGCGATA (*Eco*RI)
4808pr-BamHI-R	CGggatccCAGCCGTACTTAGCCATCTC (*Bam*HI)
4806pr-EcoRI-F	CGgaattcCTCCATACGATGTCCTCGACT (*Eco*RI)
4806pr-BamHI-R	CGggatccGCGTATTTCGCTTCCTTTGC (BamHI)
KmPstI-F	CGCGCGCctgcagTGTGTCTCAAAATC (*Pst*I)
KmPstI-R	CGCGCGCctgcagTTTAGAAAAACTCATCG (*Pst*I)
4801**RT**1	GGTGTTCTTCGCCAGATCCA
4801**RT**2	CTGCTGGACAACAAGCCCTA
4803**RT**1	CTGCGCTTGTGTTTGCTCTT
4803**RT**2	GTTCTACATGCTGGTGCCCT
4807**RT**1	CATTTTGCTTTCGTGGGCCA
4807**RT**2	TGTTGTCCATCCTGTCCAGC
phzI**RT**1	CTACCTCCTGGCGTTCAATG
phzI**RT**2	GAAGCGAGTCATTTCCCAGA
phzR**RT**1	CGCAAGGATAATCCCATCAG
phzR**RT**2	CACATTCCCTACCGCTGAAC
pip**RT**1	AAAAGACCCGCGAGAACATT
pip**RT**2	ACGTACAGCTGCTCCTTGCT
rpeB**RT**1	CATCCTTCTGGTCGAAGACG
rpeB**RT**2	AGGTCGAGAATCACCAGGTC
rpoD**RT**1	ACGTCCTGAGCGGTTACATC
rpoD**RT**2	CTTTCGGCTTCTTCTTCGTC
rpoS**RT**1	ATCAGTGGCTTTCCGAATTG
rpoS**RT**2	GACCTTCGACCTGGATCTGA

Note: Lowercase indicates nucleotides within restriction sites added to the primer for cloning purposes. The type of restriction enzymes sites at the end of primers is indicated. RT in bold indicates primers used for qRT-PCR (using the designation RT1, RT2 for forward and reverse, respectively).

Supplemental Table 2 | Amino acid identity of PcsR2 with other LuxR family regulators

Protein Tag or Protein	Strains	% Identity
C4K25_RS24230	*P. chlororaphis* ATCC 17415	100.0
A3218_RS18615	*P. chlororaphis* isolate 189	100.0
PCHL3084_RS24535	*P. chlororaphis* subsp. *aureofaciens* 30-84	100.0
C4K26_RS24620	*P. chlororaphis* TAMOak81	100.0
BLU44_RS02205	*P. chlororaphis* ATCC 13985	99.7
C4K04_RS26015	*P. chlororaphis* B25	99.7
BLU06_RS10355	*P. chlororaphis* subsp. *piscium* DSM 21509	99.7
CXP47_RS24680	*P. chlororaphis* Lzh-T5	99.7
PchlO6_5060	*P. chlororaphis* O6	99.7
EY04_RS24920	*P. chlororaphis* PA23	99.7
C4K23_RS24070	*P. chlororaphis* Pb-St2	99.7
PCAU_RS24800	*P. chlororaphis* StFRB508	99.7
C4K20_RS25530	*P. chlororaphis* subsp. *aurantiaca* CW2	99.7
C4K17_RS26260	*P. chlororaphis* subsp. *aurantiaca* DSM 19603	99.7
JM49_RS06140	*P. chlororaphis* subsp. *aurantiaca* JD37	99.7
C4K19_RS24980	*P. chlororaphis* subsp. *aurantiaca* M71	99.7
C4K10_RS24990	*P. chlororaphis* subsp. *aureofaciens* 66	99.7
C4K11_RS24555	*P. chlororaphis* subsp. *aureofaciens* C50	99.7
C4K09_RS24480	*P. chlororaphis* subsp. *aureofaciens* ChPhzS23	99.7
C4K07_RS25360	*P. chlororaphis* subsp. *aureofacien*s ChPhzS24	99.7
C4K06_RS25320	*P. chlororaphis* subsp. *aureofaciens* ChPhzTR18	99.7
C4K12_RS25290	*P. chlororaphis* subsp. *aureofaciens* ChPhzTR36	99.7
C4K05_RS25645	*P. chlororaphis* subsp. *aureofaciens* ChPhzTR38	99.7
C4K08_RS25855	*P. chlororaphis* subsp. *aureofaciens* ChPhzTR39	99.7
C4K13_RS26055	*P. chlororaphis* subsp. *aureofaciens* DSM 6698	99.7
C4K38_RS26145	*P. chlororaphis* subsp. *piscium* DSM 21509	99.7
C4K18_4867	*P. chlororaphis* subsp. *aurantiaca* k27	99.7
C4K24_RS24590	*P. chlororaphis* subsp. *aurantiaca* M12	99.3
C4K16_RS24955	*P. chlororaphis* subsp. *aurantiaca* PCM 2210	99.3
C4K15_RS25095	*P. chlororaphis* subsp. *aurantiaca* Q16	99.3
C4K27_RS25250	*P. chlororaphis* subsp. *chlororaphis* DSM 50083	99.3
EL332_RS12475	*P. chlororaphis* NCTC7357	99.0
C4K22_RS25625	*P. chlororaphis* subsp. *aurantiaca* 449	99.0
C4K21_RS25635	*P. chlororaphis* subsp. *aurantiaca* 464	99.0
C4K14_RS27040	*P. chlororaphis* subsp. *aureofaciens* P2	99.0
C4K37_RS26530	*P. chlororaphis* subsp. *piscium* ATCC 17411	99.0
C4K36_RS26520	*P. chlororaphis* subsp. *piscium* ATCC 17809	99.0
C4K31_RS25185	*P. chlororaphis* subsp. *piscium* ChPhzS135	99.0
C4K29_RS26345	*P. chlororaphis* subsp. *piscium* ChPhzS140	99.0
C4K28_RS25195	*P. chlororaphis* subsp. *piscium* ChPhzTR44	99.0
C4K34_RS25405	*P. chlororaphis* subsp. *piscium* DTR133	99.0
C4K33_RS24820	*P. chlororaphis* subsp. *piscium* PCL1391	99.0
C4K32_RS25175	*P. chlororaphis* subsp. *piscium* PCL1607	99.0

C4K35_RS27045	*P. chlororaphis* subsp. *piscium* SLPH10	99.0
C4K30_RS25710	*P. chlororaphis* subsp. *piscium* ToZa7	99.0
C6Q18_RS24930	*P. chlororaphis ZJU60*	99.0
PCL1606_RS06075	*P. chlororaphis* PCL1606	96.5
LuxR	*Vibrio fischeri*	22.1
LesR	*Lysobacter enzymogenes*	20.5
PipR	*Pseudomonas sp. strain GM79*	23.8
PsoR	*Pseudomonas fluorescens*	20.1
CsaR	*P. chlororaphis* subsp. *aureofaciens* 30-84	20.0
XagR	*Xanthomonas axonopodis pv. glycines*	18.6
PhzR	*P. chlororaphis* subsp. *aureofaciens* 30-84	18.2
NesR	*Sinorhizobium meliloti*	18.1
RhlR	*Pseudomonas aeruginosa* PAO1	17.8
XocR	*Xanthomonas oryzae* pv. *oryzae*	17.7
XccR	*Xanthomonas campestris* pv. *campestris*	17.2
OryR	*Xanthomonas oryzae* pv. *oryzae*	17.2
LasR	*Pseudomonas aeruginosa* PAO1	17.1
TraR	*Agrobacterium tumefaciens*	15.0

Note: *P. chlororaphis* strains used for comparison with PcsR2 are in black; representative QS LuxR proteins are in blue; LuxR solo proteins from plant-associated bacteria are in red.

REFERENCES

Biessy, A., Novinscak, A., Blom, J., Léger, G., Thomashow, L.S., Cazorla, F.M., et al. (2019). Diversity of phytobeneficial traits revealed by whole-genome analysis of worldwide-isolated phenazine-producing *Pseudomonas* spp. *Environmental Microbiology* 21(1)**,** 437-455. doi: 10.1111/1462-2920.14476.
